# Supplementary material for: The Association between PTPN22 SNPs and susceptibility to type 1 diabetes: An updated meta-analysis
Source: PLoS One. 2025 Apr 16;20(4):e0321624. doi: 10.1371/journal.pone.0321624 (PMC12002458; doi:10.1371/journal.pone.0321624)
Supplement: S1 Table — (DOCX) [file pone.0321624.s001.docx]

**Supplemental Table 1 Scale for quality assessment of molecular association studies of T1D**

| **Criterion** | **Score** |
| --- | --- |
| **Source of case** |  |
| Selected from population or diabetes registry | 2 |
| Selected from hospital | 1 |
| Not described | 0 |
| **Source of control** |  |
| Population-based | 2 |
| Hospital-based | 1 |
| Not described | 0 |
| **Ascertainment of T1D** |  |
| Diagnosis confirmed by endocrinologist and/or lab tests (e.g. C-peptide levels, autoantibodies) | 2 |
| Not described | 0 |
| **Family history of T1D** |  |
| Positive family history of T1D | 2 |
| Negative family history of T1D not described | 0 |
| **Ascertainment of control** |  |
| Controls were tested to screen out T1D | 2 |
| Controls were subjects who did not report T1D, no objective testing | 1 |
| Not described | 0 |
| **Matching** |  |
| Controls matched with cases by age and sex | 2 |
| Controls matched with cases only by age or sex | 1 |
| Not matched or not described | 0 |
| **Genotyping examination** |  |
| Genotyping done blindly and quality control | 2 |
| Only genotyping done blindly or quality control | 1 |
| Unblinded and without quality control | 0 |
| **HWE** |  |
| HWE in the control group | 2 |
| HWD in the control group | 0 |
| **Association assessment** |  |
| Assess association between genotypes and T1D with appropriate statistics and adjustment for confounders | 2 |
| Assess association between genotypes and T1D with appropriate statistics without adjustment for confounders | 1 |
| Inappropriate statistics used | 0 |
| **Total sample size** |  |
| >1000 | 3 |
| 500-1000 | 2 |
| 200-500 | 1 |
| <200 | 0 |

T1D: Type 1 diabetes; HWE: Hardy-Weinberg equilibrium; Hardy-Weinberg disequilibrium.
