# Supplementary material for: The Association between PTPN22 SNPs and susceptibility to type 1 diabetes: An updated meta-analysis
Source: PLoS One. 2025 Apr 16;20(4):e0321624. doi: 10.1371/journal.pone.0321624 (PMC12002458; doi:10.1371/journal.pone.0321624)
Supplement: S2 Table — (DOCX) [file pone.0321624.s002.docx]

**Supplemental Table 2 Quality assessment of included studies for assessing the quality of case control studies.**

| **First**  **Author/Year** | **Source of case** | **Source of control** | | **Diagnostic criteria** | | **Family history** | **Ascertainment of control** | **Matching** | **Genotyping examination** | **HWE** | **Association assessment** | **Total sample size** | **Quality**  **Score**  **(Total**  **score)** |
| --- | --- | --- | --- | --- | --- | --- | --- | --- | --- | --- | --- | --- | --- |
| Smyth et al.2004 | 2 | 2 | 0 | | 2 | | 2 | 0 | 1 | 2 | 0 | 2 | 13 |
| Bottini et al.2004 | 1 | 2 | 0 | | 0 | | 1 | 2 | 2 | 2 | 2 | 2 | 14 |
| Bottini et al.2004 | 1 | 2 | 0 | | 0 | | 1 | 2 | 2 | 2 | 2 | 1 | 13 |
| Zheng et al. 2005 | 2 | 2 | 2 | | 2 | | 2 | 0 | 0 | 2 | 1 | 3 | 16 |
| Kahles et al. 2005 | 2 | 2 | 2 | | 0 | | 1 | 1 | 0 | 2 | 2 | 1 | 13 |
| Gomez et al. 2005 | 2 | 2 | 0 | | 0 | | 2 | 1 | 0 | 2 | 0 | 1 | 10 |
| Zhernakova et al. 2005 | 2 | 2 | 0 | | 2 | | 0 | 1 | 0 | 2 | 0 | 2 | 11 |
| Hermann et al. 2006 | 1 | 1 | 2 | | 2 | | 1 | 2 | 0 | 2 | 2 | 3 | 16 |
| Fedetz et al.2006 | 1 | 2 | 2 | | 0 | | 2 | 2 | 0 | 2 | 1 | 2 | 14 |
| Steck et al.2006 | 1 | 2 | 0 | | 0 | | 2 | 1 | 2 | 2 | 2 | 3 | 15 |
| Chelala et al.2006 | 2 | 2 | 0 | | 2 | | 0 | 1 | 0 | 2 | 0 | 3 | 12 |
| Santiago et al.2007 | 2 | 2 | 2 | | 0 | | 1 | 2 | 2 | 2 | 2 | 2 | 17 |
| Nielsen et al.2007 | 2 | 2 | 0 | | 0 | | 1 | 2 | 2 | 2 | 0 | 2 | 13 |
| Cinek et al.2007 | 2 | 2 | 2 | | 0 | | 2 | 2 | 2 | 2 | 0 | 2 | 16 |
| Cinek et al.2007 | 2 | 2 | 2 | | 0 | | 2 | 2 | 2 | 2 | 0 | 1 | 15 |
| Baniasadi et al.2008 | 2 | 2 | 2 | | 0 | | 1 | 2 | 2 | 2 | 0 | 1 | 14 |
| Douroudis et al.2008 | 1 | 1 | 2 | | 0 | | 1 | 2 | 2 | 2 | 1 | 1 | 15 |
| Dultz et al.2008 | 2 | 2 | 2 | | 0 | | 1 | 2 | 2 | 2 | 0 | 0 | 13 |
| Smyth et al.2008 | 2 | 2 | 0 | | 0 | | 1 | 1 | 0 | 2 | 0 | 3 | 11 |
| Korolija et al.2009 | 2 | 2 | 0 | | 0 | | 1 | 2 | 0 | 2 | 0 | 1 | 10 |
| Lavrikova et al.2009 | 2 | 2 | 0 | | 2 | | 1 | 2 | 0 | 2 | 0 | 1 | 12 |
| Fichna et al.2010 | 2 | 2 | 2 | | 0 | | 2 | 2 | 0 | 2 | 0 | 1 | 13 |
| Kordonouri et al.2010 | 2 | 2 | 2 | | 0 | | 1 | 2 | 2 | 2 | 0 | 1 | 14 |
| Taniyama et al.2010 | 2 | 2 | 2 | | 0 | | 1 | 2 | 0 | 2 | 0 | 1 | 12 |
| Chagastelles et al.2010 | 1 | 1 | 2 | | 0 | | 1 | 2 | 2 | 2 | 2 | 1 | 14 |
| Zhebrun et al.2011 | 2 | 2 | 2 | | 0 | | 1 | 2 | 0 | 0 | 2 | 1 | 12 |
| Liu et al.2012 | 2 | 2 | 2 | | 0 | | 1 | 2 | 2 | 0 | 0 | 1 | 12 |
| Kisand et al.2012 | 1 | 1 | 2 | | 0 | | 1 | 2 | 2 | 2 | 2 | 1 | 14 |
| Sharma et al.2012 | 1 | 2 | 2 | | 0 | | 1 | 2 | 2 | 2 | 2 | 1 | 15 |
| Giza et al.2013 | 2 | 2 | 0 | | 2 | | 2 | 2 | 2 | 2 | 0 | 1 | 15 |
| Hadzija et al.2013 | 2 | 2 | 2 | | 0 | | 2 | 2 | 2 | 2 | 0 | 1 | 15 |
| Almasi et al.2014 | 2 | 2 | 2 | | 0 | | 1 | 2 | 0 | 2 | 0 | 1 | 12 |
| kumar et al.2014 | 1 | 2 | 0 | | 0 | | 1 | 2 | 1 | 2 | 2 | 1 | 12 |
| Min et al.2014 | 2 | 2 | 2 | | 0 | | 1 | 2 | 2 | 2 | 0 | 0 | 13 |
| Zouidi et al.2014 | 1 | 1 | 2 | | 2 | | 1 | 1 | 2 | 2 | 2 | 1 | 15 |
| Liu et al.2015 | 1 | 2 | 0 | | 0 | | 1 | 2 | 2 | 2 | 2 | 1 | 13 |
| Pawlowicz et al.2017 | 2 | 2 | 2 | | 0 | | 1 | 2 | 2 | 2 | 0 | 1 | 14 |
| Abbasi et al.2017 | 2 | 2 | 2 | | 0 | | 1 | 1 | 2 | 2 | 0 | 0 | 12 |
| Heneberg et al.2018 | 1 | 1 | 2 | | 0 | | 1 | 2 | 2 | 2 | 2 | 2 | 15 |
| Alswat et al.2018 | 1 | 1 | 2 | | 0 | | 2 | 2 | 2 | 0 | 2 | 2 | 14 |
| El Fotoh et al.2019 | 1 | 1 | 2 | | 2 | | 2 | 2 | 2 | 2 | 2 | 1 | 17 |
| Rochmah et al.2023 | 1 | 1 | 2 | | 2 | | 1 | 2 | 2 | 0 | 2 | 0 | 13 |
| Zak et al.2023 | 1 | 2 | 2 | | 0 | | 2 | 1 | 2 | 2 | 2 | 0 | 14 |
