# Supplementary material for: The Association between PTPN22 SNPs and susceptibility to type 1 diabetes: An updated meta-analysis
Source: PLoS One. 2025 Apr 16;20(4):e0321624. doi: 10.1371/journal.pone.0321624 (PMC12002458; doi:10.1371/journal.pone.0321624)
Supplement: S3 Table — (DOCX) [file pone.0321624.s003.docx]

**Supplemental Table 3 Included studies of PTPN22 polymorphisms in T1D within the meta-analyses.**

| **No.** | | **First Author/Year** | | **Country** | | **Eligible research studies of PTPN22 rs2476601** | | | | |
| --- | --- | --- | --- | --- | --- | --- | --- | --- | --- | --- |
|  |  |  |  |  |  | **All studies** | | | | |
|  |  |  |  |  |  | **This study** | **Peng et al. 2012** | **tang et al. 2012** | **Lee et al. 2013** | **xuan et al. 2013** |
| 1 | Smyth et al.2004 | | U.K. | | C | | C | C | C | C |
| 2 | Bottini et al.2004 | | U.S | | C | | C | C | - | C |
| 3 | Bottini et al.2004 | | Italian | | C | | C | C | - | C |
| 4 | Zheng et al. 2005 | | U.S | | C | | M | M | - | C |
| 5 | Kahles et al. 2005 | | German | | C | | C | C | - | C |
| 6 | Gomez et al. 2005 | | Colombian | | M | | M | M | - | C |
| 7 | Zhernakova et al. 2005 | | Netherlands | | C | | C | C | C | C |
| 8 | Ladner et al. 2005 | | Unidentified | | U(NR) | | - | - | C | - |
| 9 | Hermann et al. 2006 | | Finland | | C | | C | - | - | C |
| 10 | Fedetz et al.2006 | | Ukraine | | C | | C | C | - | C |
| 11 | Steck et al.2006 | | U.S | | C | | M | M | - | C |
| 12 | Chelala et al.2006 | | France | | M | | C | C | - | C |
| 13 | Santiago et al.2007 | | Spain | | C | | C | C | - | C |
| 14 | Nielsen et al.2007 | | Danish | | C | | C |  | - | C |
| 15 | Cinek et al.2007 | | Czech | | C | | C | C | - | C |
| 16 | Cinek et al.2007 | | Azeri | | C | | A | C | - | C |
| 17 | Petrone et al.2007 | | Italian | | C(NR) | | C | C | - | C |
| 18 | Chelala et al.2007 | | French, U.S, and Danish | | M(NR) | | - | - | C | - |
| 19 | Baniasadi et al.2008 | | India | | A | | A | A | - | A |
| 20 | Douroudis et al.2008 | | Estonia | | C | | C | C | - | C |
| 21 | Dultz et al.2008 | | German | | C | | C | C | - | C |
| 22 | Smyth et al.2008 | | U.K. | | C | | C | - | - | C |
| 23 | Saccucci et al.2008 | | Italy | | C(NR) | | - | - | - | C |
| 24 | Cervin et al.2008 | | Sweden | | C(NR) | | - | - | - | C |
| 25 | Zoledziewska et al.2008 | | Sardinian | | C(NR) | | - | - | C | - |
| 26 | Korolija et al.2009 | | Croatia | | C | | C | C | - | C |
| 27 | Stene et al.2009 | | Norway | | C(NR) | | C | - | - | C |
| 28 | Lavrikova et al.2009 | | Russia | | C | | - | C | C | - |
| 29 | Fichna et al.2010 | | Polish | | C | | C | C | - | C |
| 30 | Kordonouri et al.2010 | | German | | C | | C | - | - | C |
| 31 | Chagastelles et al.2010 | | Brazilian | | C | | M | M | - | C |
| 32 | Klinker et al.2010 | | Finland | | C(NR) | | - | - | - | C |
| 33 | Zhebrun et al.2011 | | Russia | | C(HWD) | | C | C | - | C |
| 34 | Liu et al.2012 | | China | | A(HWD) | | - | A | - | - |
| 35 | Kisand et al.2012 | | Estonia | | C | | - | - | - | C |
| 36 | Giza et al.2013 | | Greek | | C | | - | - | - | - |
| 37 | Hadzija et al.2013 | | Bosnia and Herzegovina | | C | | - | - | - | - |
| 38 | Almasi et al.2014 | | Iran | | C | | - | - | - | - |
| 39 | kumar et al.2014 | | India | | A | | - | - | - | - |
| 40 | Min et al.2014 | | China | | A | | - | - | - | - |
| 41 | Liu et al.2015 | | China | | A | | - | - | - | - |
| 42 | Pawlowicz et al.2017 | | Poland | | M | | - | - | - | - |
| 43 | Heneberg et al.2018 | | Czech | | C | | - | - | - | - |
| 44 | Alswat et al.2018 | | Saudi | | A(HWD) | | - | - | - | - |
| 45 | El Fotoh et al.2019 | | Egyptia | | M | | - | - | - | - |
| 46 | Rochmah et al.2023 | | Indonesia | | M(HWD) | | - | - | - | - |
| 47 | Zak et al.2023 | | America | | C | | - | - | - | - |

A, Asian; I, Indian; Af, African; C, Caucasion; M, Mixed; U, unidentified; HWD = Hardy-Weinberg Disequilibrium; NR: Complete data not Reported.
