# Supplementary material for: The Association between PTPN22 SNPs and susceptibility to type 1 diabetes: An updated meta-analysis
Source: PLoS One. 2025 Apr 16;20(4):e0321624. doi: 10.1371/journal.pone.0321624 (PMC12002458; doi:10.1371/journal.pone.0321624)
Supplement: S4 Table — (DOCX) [file pone.0321624.s004.docx]

**Supplemental Table 4** **Risk Allele Frequency Comparisons (Current Study, HapMap, 1000 Genomes).**

| Gene | SNP | Population | Risk Allele | HapMap | | 1000 genomes | | Case | | Controls | |
| --- | --- | --- | --- | --- | --- | --- | --- | --- | --- | --- | --- |
|  |  |  |  | n | RAF | n | RAF | n | RAF | n | RAF |
| PTPN22 | rs2476601 | European | T | 172 | 7.0% | 1006 | 9.4% | 15918 | 20.6% | 19349 | 13.4% |
| PTPN22 | rs2476601 | Asian | T | 250 | 1.6% | 2210 | 0.7% | 988 | 8.4% | 924 | 3.1% |
| PTPN22 | rs2476601 | African | T | 682 | 0.4% | 1322 | 0.3% | - | - | - | - |
| PTPN22 | rs2476601 | American | T | 756 | 4.9% | 694 | 3.6% | - | - | - | - |
| PTPN22 | rs1310182 | European | C | - | - | 1006 | 55.4% | 519 | 52.1% | 451 | 47.8% |
| PTPN22 | rs1310182 | Asian | C | - | - | 1986 | 58.1% | 323 | 57.3% | 350 | 69.3% |
| PTPN22 | rs1310182 | African | C | - | - | 1322 | 66.7% | - | - | - | - |
| PTPN22 | rs1310182 | American | C | - | - | 694 | 41.9% | - | - | - | - |

RAF: Risk Allele Frequency.
