# Supplementary material for: The Association between PTPN22 SNPs and susceptibility to type 1 diabetes: An updated meta-analysis
Source: PLoS One. 2025 Apr 16;20(4):e0321624. doi: 10.1371/journal.pone.0321624 (PMC12002458; doi:10.1371/journal.pone.0321624)
Supplement: Attachment — Submitted filename: PONE-D-24-50318.pdf [file pone.0321624.s008.pdf]

PLOS ONE

The Association between PTPN22 SNPs and Susceptibility to Type 1 Diabetes: An updated meta-analysis

--Manuscript Draft--

**Manuscript Number:**	PONE-D-24-50318
**Article Type:**	Research Article
**Full Title:**	The Association between PTPN22 SNPs and Susceptibility to Type 1 Diabetes: An updated meta-analysis
**Short Title:**	The Association between PTPN22 SNPs and Susceptibility to Type 1 Diabetes: An updated meta-analysis
**Corresponding Author:**	Fei-fei Wu<br>chang zhi yi xue yuan fu shu he ping yi yuan: Changzhi Medical College Affiliated Heping Hospital<br>Changzhi, CHINA
**Keywords:**	PTPN22, polymorphism, T1D, Meta-analysis
**Abstract:**	Type 1 diabetes (T1D) represents a significant global health concern, characterized by the autoimmune destruction of insulin-producing pancreatic β-cells, resulting in lifelong dependence on insulin therapy Although genetic prdisposition represents a pivotal element in the pathogenesis of T1D, environmental factors also exert a contributory influence on its onset and progression. Recent research has identified a number of genetic polymorphisms, particularly in the protein tyrosine phosphatase non-receptor 22 gene (PTPN22), that are strongly associated with an increased risk of T1D and may serve as potential biomarkers for early diagnosis and prevention. However, the findings regarding the relationship between PTPN22 SNPs and T1D susceptibility have been inconsistent. This study presents an updated meta-analysis of two key PTPN22 polymorphic loci — rs2476601 (C1858T) and rs1310182 (A852G) — with the aim of clarifying their associations with T1D. The analysis yielded significant results with regard to the association between PTPN22 rs2476601 and an increased risk of T1D. In contrast, no significant correlations were identified for rs1310182. These findings highlight the potential utility of PTPN22 rs2476601 as a marker for T1D susceptibility, offering insights for the development of early intervention strategies. Further research is required to validate these associations and enhance our understanding of the genetic factors involved in T1D pathogenesis.
**Order of Authors:**	Yu - Su
	Xue - Li
	Pei-dong - Wu
	Yu-Long Zhang
	Peng-fei Fang
	Xiao-feng He
	Fei-fei Wu
**Additional Information:**	
**Question**	**Response**
**Financial Disclosure**<br><br>Enter a financial disclosure statement that describes the sources of funding for the work included in this submission. Review the submission guidelines for detailed requirements. View published research articles from *PLOS ONE* for specific	The author(s) received no specific funding for this work.

Powered by Editorial Manager® and ProduXion Manager® from Aries Systems Corporation

examples.<br><br>This statement is required for submission and **will appear in the published article** if the submission is accepted. Please make sure it is accurate.<br><br>**Funded studies**<br>Enter a statement with the following details:<br>• Initials of the authors who received each award<br>• Grant numbers awarded to each author<br>• The full name of each funder<br>• URL of each funder website<br>• Did the sponsors or funders play any role in the study design, data collection and analysis, decision to publish, or preparation of the manuscript?<br><br>Did you receive funding for this work?	
**Competing Interests**<br><br>Use the instructions below to enter a competing interest statement for this submission. On behalf of all authors, disclose any competing interests that could be perceived to bias this work—acknowledging all financial support and any other relevant financial or non-financial competing interests.<br><br>This statement is required for submission and **will appear in the published article** if the submission is accepted. Please make sure it is accurate and that any funding sources listed in your Funding Information later in the submission form are also declared in your Financial Disclosure statement.<br><br>View published research articles from *PLOS ONE* for specific examples.	There are no conflicts of interest among the authors.

Powered by Editorial Manager® and ProduXion Manager® from Aries Systems Corporation

**NO authors have competing interests**<br><br>Enter: *The authors have declared that no competing interests exist.*<br><br>**Authors with competing interests**<br><br>Enter competing interest details beginning with this statement:<br><br>*I have read the journal's policy and the authors of this manuscript have the following competing interests: [insert competing interests here]*<br><br>* typeset	
**Ethics Statement**<br><br>Enter an ethics statement for this submission. This statement is required if the study involved:<br><br>• Human participants<br>• Human specimens or tissue<br>• Vertebrate animals or cephalopods<br>• Vertebrate embryos or tissues<br>• Field research<br><br>Write "N/A" if the submission does not require an ethics statement.<br><br>General guidance is provided below. Consult the submission guidelines for detailed instructions. **Make sure that all information entered here is included in the Methods section of the manuscript.**	N/A

Powered by Editorial Manager® and ProduXion Manager® from Aries Systems Corporation

**Format for specific study types**<br><br>**Human Subject Research (involving human participants and/or tissue)**<br>• Give the name of the institutional review board or ethics committee that approved the study<br>• Include the approval number and/or a statement indicating approval of this research<br>• Indicate the form of consent obtained (written/oral) or the reason that consent was not obtained (e.g. the data were analyzed anonymously)<br><br>**Animal Research (involving vertebrate animals, embryos or tissues)**<br>• Provide the name of the Institutional Animal Care and Use Committee (IACUC) or other relevant ethics board that reviewed the study protocol, and indicate whether they approved this research or granted a formal waiver of ethical approval<br>• Include an approval number if one was obtained<br>• If the study involved *non-human primates*, add *additional details* about animal welfare and steps taken to ameliorate suffering<br>• If anesthesia, euthanasia, or any kind of animal sacrifice is part of the study, include briefly which substances and/or methods were applied<br><br>**Field Research**<br><br>Include the following details if this study involves the collection of plant, animal, or other materials from a natural setting:<br>• Field permit number<br>• Name of the institution or relevant body that granted permission	
**Data Availability**<br><br>Authors are required to make all data underlying the findings described fully available, without restriction, and from the time of publication. PLOS allows rare exceptions to address legal and ethical concerns. See the PLOS Data Policy and FAQ for detailed information.	Yes - all data are fully available without restriction

A Data Availability Statement describing where the data can be found is required at submission. Your answers to this question constitute the Data Availability Statement and **will be published in the article**, if accepted.<br><br>**Important:** Stating 'data available on request from the author' is not sufficient. If your data are only available upon request, select 'No' for the first question and explain your exceptional situation in the text box.<br><br>Do the authors confirm that all data underlying the findings described in their manuscript are fully available without restriction?	
**Describe where the data may be found in full sentences. If you are copying our sample text, replace any instances of XXX with the appropriate details.**<br><br>• If the data are **held or will be held in a public repository**, include URLs, accession numbers or DOIs. If this information will only be available after acceptance, indicate this by ticking the box below. For example: *All XXX files are available from the XXX database (accession number(s) XXX, XXX.).*<br>• If the data are all contained **within the manuscript and/or Supporting Information files**, enter the following: *All relevant data are within the manuscript and its Supporting Information files.*<br>• If neither of these applies but you are able to provide **details of access elsewhere**, with or without limitations, please do so. For example:<br><br>*Data cannot be shared publicly because of [XXX]. Data are available from the XXX Institutional Data Access / Ethics Committee (contact via XXX) for researchers who meet the criteria for access to confidential data.*<br><br>*The data underlying the results presented in the study are available from (include the name of the third party*	We gratefully acknowledge the contributors to all the original studies included in this meta-analysis.

Powered by Editorial Manager® and ProduXion Manager® from Aries Systems Corporation

and contact information or URL).<br>• This text is appropriate if the data are owned by a third party and authors do not have permission to share the data.<br><br>* typeset	
Additional data availability information:	

Manuscript Click here to access/download;Manuscript;Manuscript.docx

The Association between PTPN22 SNPs and Susceptibility to Type 1 Diabetes: An updated meta-analysis

Yu Su[1], Xue LI[1], Pei-dong Wu[2], Yu-long Zhang[3], Peng-fei Fang[4], Fei-fei Wu[1*], Xiao-feng He[5*]

[1] *Department of Endocrinology, Heping Hospital Affiliated to Changzhi Medical College, Shanxi Province, Changzhi, China*

[2] *Shanxi Medical University, Shanxi Province, Taiyuan, China*

[3] *Department of Oncology, Handan First Hospital, Hebei Province, Handan, China*

[4] *Department of Cardiology, Henan Province, Zhoukou, China*

[5] *Institute of Evidence-Based Medicine, Heping Hospital Affiliated to Changzhi Medical College, Shanxi Province, Changzhi, China*

***Corresponding author:** Fei-fei Wu (E-mail: h4377343@ 163.com) and Xiao-feng He (E-mail: 393120823@ qq.com)

Abstract

Type 1 diabetes (T1D) represents a significant global health concern, characterized by the autoimmune destruction of insulin-producing pancreatic β-cells, resulting in lifelong dependence on insulin therapy Although genetic prdisposition represents a pivotal element in the pathogenesis of T1D, environmental factors also exert a contributory influence on its onset and progression. Recent research has identified a number of genetic polymorphisms, particularly in the protein tyrosine phosphatase non-receptor 22 gene (PTPN22), that are strongly associated with an increased risk of T1D and may serve as potential biomarkers for early diagnosis and prevention. However, the findings regarding the relationship between PTPN22 SNPs and T1D susceptibility have been inconsistent. This study presents an updated meta-analysis of two key PTPN22 polymorphic loci — rs2476601 (C1858T) and rs1310182 (A852G) — with the aim of clarifying their associations with T1D. The analysis yielded significant results with regard to the association between PTPN22 rs2476601 and an increased risk of T1D. In contrast, no significant correlations were identified for rs1310182. These findings highlight the potential utility of PTPN22 rs2476601 as a marker for T1D susceptibility, offering insights for the development of early intervention strategies. Further research is required to validate these associations and enhance our understanding of the genetic factors involved in T1D pathogenesis.

Keywords: PTPN22, polymorphism, T1D, Meta-analysis

Introduction

T1D is a complex autoimmune disease that results in the destruction of pancreatic β - cells, necessitating lifelong insulin therapy [1]. The pathogenesis of this disease is influenced by both genetic predisposition and environmental factors, with a rising global incidence. In 2017, approximately 230,000 new cases were reported, resulting in a total of approximately 9 million cases worldwide. In addition to the HLA region, PTPN22 gene is now recognized as one of the strongest genetic risks for promoting the development of autoimmunity [2].

Human genetic polymorphisms significantly impact disease susceptibility, clinical manifestations, and drug response variability. Polymorphisms in the PTPN22 gene have been associated with an increased risk of developing various autoimmune disorders, including T1D. PTPN 22 plays a pivotal role in T cell signaling and immune response, functioning as a key desensitization node with multiple validated substrates and several putative substrates [3]. These variations, such as single nucleotide polymorphisms, can alter the efficacy and toxicity of chemotherapeutic drugs, leading to inter-individual difference [4]. PTPN22 encodes a lymphatic tyrosine phosphatase (LYP) that negatively regulates T-cell receptor (TCR) signaling. The R620W substitution

impairs LYP's interaction with C-terminal Src Kinase (CSK), reducing LYP's inhibitory effect and enhancing TCR signaling [5]. This alteration in signaling pathways increases susceptibility to autoimmune diseases by affecting T cell tolerance and response. The PTPN22 rs2476601 polymorphism was first linked to T1D in American and Italian populations by Bottini et al. in 2004 [6], and subsequent studies confirmed this association in Caucasian groups [7–10]. In contrast, the rs2476601 polymorphism was initially reported as non-polymorphic in Asian populations [11]. However, a recent study by Kawasaki et al. suggested that the promoter subregion 1123G/C (rs2488457) of the PTPN22 gene is associated with acute-onset T1D in the Japanese population, linking this polymorphism to rs2476601 in Caucasians [12]. Some studies propose that the association of 1123G/C (rs2488457) with autoimmune diseases results from linkage to C1858T (rs2476601), with 1858T identified as the true susceptibility allele [11].

Similarly, rs1310182 is an intronic sub-polymorphism situated within a putative transcription factor binding site of the PTPN 22 gene, yet it also encompasses the joint-associated protein complex 4 subunit beta 1 (AP4B1) antisense RNA 1 gene [13–16]. Initially, it was demonstrated to be unrelated to T1D [17], which challenges the prevailing notion that it is pertinent to autoimmune disease. However, later studies reported varied associations, with inconsistency in identifying the specific allele related to the disease. For example, in Armenians, the rs1310182 T allele was positively associated with T1D, while the CC genotype was negatively associated [18]. These associations differ across ethnic groups, as seen in studies from Japan, the UAE, Iran, and Tunisia [14,19–21].

Accordingly, we selected two SNPs in the PTPN22 gene, rs2476601 and rs11310182 for this study. Although these polymorphisms have been examined in 64 studies, the association between these SNPs and T1D risk remains unclear due to small sample sizes and conflicting results. Previous meta-analyses have confirmed the link between rs2476601 and T1D risk in Caucasians and American [7–10], but its association in other races is still uncertain. Additionally, rs1310182 has not been fully explored in relation to patient clinical characteristics in existing meta-analyses. To provide more robust conclusions, we conducted an updated systematic review and meta-analysis, enhancing data collection and refining statistical evaluation methods.

Materials and methods

Search strategy

A comprehensive search for relevant literature was conducted in a number of databases, including PubMed, Web of Knowledge, China Knowledge, and Wanfang. The search was carried out in strict accordance with the PRISMA criteria and the resulting literature was subsequently confirmed through a process of title and abstract screening, followed by a detailed examination of the full texts [22]. Additionally, references of identified meta-analyses and reviews were examined to ascertain whether any pertinent studies had been overlooked. The search was concluded in April 2024. No restrictions were placed on language or ethnicity. The search strategy for the English database was as follows: The search terms included "polymorphism," "variant," "variation," "mutation," "SNP," "genome-wide association study," "genetic association study," "genotype," and "allele." These were combined with the search terms "type 1 diabetes," "type 1 diabetes mellitus," "type 1 diabetes mellitus," and "diabetes mellitus," as well as "T1D." The search was further refined by including the search terms "PTPN22" and "protein tyrosine phosphatase nonreceptor 22." 10-Methylenetetrahydrofolate reductase. The Chinese search terms are as follows: The subject terms used to search the Chinese database were "protein tyrosine phosphatase 22," "PTPN22," "gene polymorphism," and "type 1 diabetes mellitus."

Selection criteria

Inclusion criteria: (1) Case-control or cohort studies; (2) Correlation studies on the relationship between polymorphisms in the PTPN22 rs2476601 and/or rs1310182 genes and T1D susceptibility; (3) Genotype

frequencies corresponding to genetic polymorphisms in the case and control groups can be obtained from the literature; (4) The study must be administrated in human samples.

Exclusion criteria: (1) Studies with missing or duplicated data; (2) reviews, letters, and case reports; (3) Studies with type 1 diabetes included in the case group; (4) studies with poorly described data.

Data extraction

To ensure the accuracy of the extracted information, each study meeting the inclusion and exclusion criteria was independently assessed by two authors. Disagreements were resolved through discussion, and if unresolved, the corresponding author re-extracted and validated the data. For studies with insufficient detail, efforts were made to contact the original authors for clarification. Incomplete data studies were excluded, retaining only high-quality studies among duplicates. Key information extracted included: first author's last name, publication year, country, ethnicity (Asian, Caucasian, African, Indian, mixed), sample size, control source (hospital-based or population-based), type of matching, blinding and quality control of genotyping, and statistical adjustments for assessing associations between genotypes and T1D. Details are summarized in **Tables 1** and **2**.

Table 1. Characteristics of studies on rs2476601 included in the meta-analysis.

First author/Year	Country	Ethnicity	SC	Type of control	Sample size	Genotypes distribu (rs2476		
						Cases		
						CC	CT	TT
Smyth et al.2004 [23]	U.K.	Caucasian	PB	Normoglycemic controls	294/395	661	222	18
Bottini et al.2004 [6]	U.S	Caucasian	HB	Healthy controls	294/395	193	90	11
Bottini et al.2004 [6]	Italian	Caucasian	HB	Healthy controls	174/214	158	15	1
Zheng et al. 2005 [24]	U.S	Caucasian	PB	Normoglycemic controls	396/1178	290	97	9
Kahles et al. 2005 [25]	German	Caucasian	PB	Healthy controls	220/239	142	71	7
Gomez et al. 2005 [26]	Colombian	Mixed	PB	Normoglycemic controls	110/308	94	38	1
Zhernakova et al. 2005 [27]	Netherlands	Caucasian	PB	NR	334/528	226	96	12
Hermann et al. 2006 [28]	Finland	Caucasian	HB	Healthy controls	546/538	316	200	30
Fedetz et al.2006 [29]	Ukraine	Caucasian	HB	Normoglycemic controls	296/242	187	93	16
Steck et al.2006 [30]	U.S	Caucasian	HB	Normoglycemic controls	690/515	482	193	15
Chelala et al.2006 [31]	France	Mixed	PB	NR	885/442	623	243	19
Santiago et al.2007 [32]	Spain	Caucasian	PB	Healthy controls	554/316	483	68	3
Nielsen et al.2007 [33]	Danish	Caucasian	PB	Healthy controls	253/354	182	61	10
Cinek et al.2007 [34]	Czech	Caucasian	PB	Normoglycemic controls	372/400	231	127	14
Cinek et al.2007 [34]	Azeri	Caucasian	PB	Normoglycemic controls	160/271	152	7	1
Baniasadi et al.2008 [35]	India	Asian	PB	Healthy controls	129/109	121	7	1
Douroudis et al.2008 [36]	Estonia	Caucasian	HB	Healthy controls	170/230	99	57	14
Dultz et al.2008 [37]	German	Caucasian	PB	Healthy controls	70/100	55	15	0
Smyth et al.2008 [38]	U.K.	Caucasian	PB	Healthy controls	8984/10930	5030	2165	239
Korolija et al.2009 [39]	Croatia	Caucasian	PB	Healthy controls	102/193	47	51	4
Lavrikova et al.2009 [40]	Russia	Caucasian	PB	Healthy controls	162/203	108	49	5
Fichna et al.2010 [41]	Polish	Caucasian	PB	Normoglycemic controls	215/236	143	64	8
Kordonouri et al.2010 [42]	German	Caucasian	PB	Healthy controls	243/209	177	58	8
Chagastelles et al.2010 [43]	Brazilian	Caucasian	HB	Healthy controls	211/241	152	56	3
Zhebrun et al.2011 [44]	Russia	Caucasian	PB	Healthy controls	150/200	99	40	11

Liu et al.2012 [45]	China	Asian	PB	Healthy controls	229/210	228	1	0
Kisand et al.2012 [46]	Estonia	Caucasian	HB	Healthy controls	154/229	88	53	13
Giza et al.2013 [47]	Greek	Caucasian	PB	Normoglycemic controls	130/135	116	13	1
Hadzija et al.2013 [48]	Bosnia and Herzegovina	Caucasian	PB	Normoglycemic controls	241/161	128	100	13
Almasi et al.2014 [49]	Iran	Caucasian	PB	Healthy controls	144/197	140	4	0
Kumar et al.2014 [50]	India	Indian	HB	Healthy controls	145/210	137	8	0
Min et al.2014 [51]	China	Asian	PB	Healthy controls	19/20	13	4	2
Liu et al.2015 [52]	China	Asian	HB	Healthy controls	239/213	195	38	6
Pawlowicz et al.2017 [53]	Poland	Mixed	PB	Healthy controls	147/327	91	44	12
Heneberg et al.2018 [13]	Czech	Caucasian	HB	Healthy controls	263/400	168	84	11
Alswat et al.2018 [54]	Saudi	Asian	HB	no family history	372/372	290	66	16
El Fotoh et al.2019 [55]	Egyptia	Mixed	HB	Normoglycemic controls	120/120	81	33	6
Rochmah et al.2023 [56]	Indonesia	Mixed	HB	Healthy controls	31/31	3	28	0
Zak et al.2023 [18]	America	Caucasian	HB	Healthy controls	96/100	87	9	0

HB = hospital-based studies, PB = population-based studies, HWE= Hardy-Weinberg equilibrium, HWD = Hardy-Weinberg Disequilibrium, NR = Not reported.

Table 2. Characteristics of studies on rs1310182 included in the meta-analysis.

First author/Year	Country	Ethnicity	SC	Type of control	Sample size	Genotypes distribution o		
						Cases		
						CC	CT	TT
Taniyama et al.2010 [14]	Japan	Asian	PB	Healthy controls	184/179	103	66	15
Sharma et al.2012 [57]	Emirati nationals	Asian	HB	Healthy controls	139/171	24	50	65
Zouidi et al.2014 [20]	Republic of Tunis	Caucasian	HB	Healthy controls	76/151	7	40	29
Abbasi et al.2017 [19]	Iran	Caucasian	PB	Healthy controls	99/100	26	40	33
Heneberg et al.2018 [13]	Czech	Caucasian	HB	Healthy controls	248/100	103	105	40
Zak et al.2023 [58]	America	Caucasian	HB	Healthy controls	96/100	13	58	25

HB = hospital-based studies, PB = population-based studies, HWE= Hardy-Weinberg equilibrium.

Quality assessment

A quality assessment scale was developed based on PRISMA guidelines, the quality reporting guidelines for observational studies, and previous meta-analyses [7–10]. Two investigators independently extracted and cross-examined data to resolve disagreements through discussion. If consensus was not reached, a third author would perform a final review. Original authors could also be contacted for clarification if needed. **Supplemental Table 1** details the quality indicators used to assess the studies. The control group was evaluated using a goodness-of-fit chi-square test, and Hardy-Weinberg equilibrium (HWE) tests were applied to studies with complete genotypic data. A p-value of less than 0.05 indicated significant bias. Studies scoring 12 or above were classified as high quality, contingent on meeting both quality scores and HWE criteria. Discrepancies in scores were evaluated by leading experts in the field.

Supplemental Table 1 Scale for quality assessment of molecular association studies of T1D

Statistical analysis

The pooled odds ratios (ORs) and corresponding 95% confidence intervals (CIs) were calculated to assess the association between PTPN22 polymorphisms and the risk of T1D, with a p-value of less than 0.05 indicating

statistical significance. The PTPN22 rs2476601 models included: (1) allele model C vs T; (2) additive model CC vs TT; (3) dominant model CC + CT vs TT; (4) recessive model CC vs CT + TT; and (5) super-dominant model CT vs CC. The PTPN22 rs1310182 models included: (1) allele model C vs T; (2) additive model CC vs TT; (3) dominant model CC + CT vs TT; (4) recessive model CC vs CT + TT; and (5) super-dominant model CT vs CC. HWE was assessed using the goodness-of-fit test, with significant disequilibrium defined as Hardy-Weinberg Disequilibrium (HWD) when P < 0.05. Subgroup analyses were performed based on characteristics such as genotyping quality, HWE status, control source, geographic region, and ethnicity. The chi-square Q-test and I^2 statistic assessed heterogeneity, with a p-value greater than 0.10 or I^2 less than 50% indicating no significant heterogeneity, prompting the use of a fixed-effects model [59]; otherwise, a random-effects model was applied [60]. If there was significant heterogeneity, we may use meta-regression analysis to search for the sources of heterogeneity. Robustness was ensured through sensitivity analyses, including the removal of individual studies, exclusion of low-quality studies and those in HWD, and selection of high-quality studies meeting HWE criteria. Begg's funnel plot and Egger's test were employed to evaluate the stability of the results [61]. In instances where publication bias was evident [62], a non-parametric 'trim-and-fill' method was employed to rectify and identify asymmetries in the funnel plot resulting from publication bias, while simultaneously estimating the true value of the quantitative composite [63]. Furthermore, the confidence of significant results was investigated using the following criteria: false positive reporting probability (FPRP), Bayesian false discovery probability (BFDP) and Venice criteria [64,65]. Significant correlations were identified as 'positive results' if they met the following statistical criteria: (1) In at least two genetic models, the p-value of the Z-test were <0.05; (2) FPRP < 0.2 and BFDP < 0.8; (3) statistical efficiency > 0.8; (4) I^2 < 50%. The aforementioned statistical analyses were conducted using STATA 12.0.

Results

Description of included studies

A total of 1,581 articles were obtained through a structured search strategy (see **Fig 1** for the search and selection process). After screening titles, abstracts, and full texts, 43 articles and 45 studies were identified for inclusion in the meta-analysis, meeting the established criteria. These studies, published between 2004 and 2023, included 19,186 cases of type 1 diabetes and 22,512 controls. Specifically, 39 studies addressed the PTPN22 rs2476601 polymorphism, while six focused on PTPN22 rs1310182. A total of 32 studies were conducted on Caucasian populations, seven on Asian populations, five on mixed-race populations, one on Indian populations, and none on African populations. To minimize the influence of low-quality studies, we assessed the quality of the included research, finding that 32 high-quality studies (scoring >12 points) examined the rs2476601 polymorphism and four focused on rs1310182. The distribution of control genotypes aligned with HWE in all but two studies. **Tables 1 - 2** show the genotype frequencies and HWE test results of rs2476601, 1310182 in relation to T1D risk.

Fig. 1 Flowchart of Literature Retrieval Selection

Meta-analysis results

Pooled analysis for PTPN22 rs2476601

This meta-analysis included 39 case-control studies (18,344 cases and 21,711 controls) that investigated the association between PTPN22 rs2476601 and T1D susceptibility. As shown in **Table 3**, a significant associations with T1D risk was observed across all genetic models compared to the control group, with the following odds ratios (ORs) and 95% confidence intervals (CIs): CC vs. TT: OR = 0.133, 95% CI = 0.087-0.203; CT vs. CC: OR = 1.777, 95% CI = 1.484-2.128; CT+TT vs. CC: OR = 0.212, 95% CI = 0.172-0.262; CT+CC vs. TT: OR = 0.341, 95% CI = 0.231-0.502; C vs. T: OR = 0.547, 95% CI = 0.426-0.701. In race-based subgroup

analyses, the PTPN22 rs2476601 gene polymorphism was significantly associated with the risk of T1D in Caucasians (CC vs. TT: OR = 0.119, 95% CI = 0.099-0.144; CT vs. CC: OR = 1.764, 95% CI = 1.553-2.004) and Asian races (CC vs. TT: OR = 0.064, 95% CI = 0.028-0.149; CT vs. CC: OR = 2.921, 95% CI = 2.027-2.207). These results were not statistically significant in mixed populations. In population-based studies, negative associations were observed when the data were stratified by source of control and Type of control. All indicate that PTPN22 polymorphisms increase the risk of T1D.

Table 3 Meta-analysis of the association of PTPN22 rs2476601 polymorphism with risk of T1D.

Variable	n (Cases/Controls)	CC vs. TT		CT vs. CC	
		OR (95% CI)	P_h/I^2(%)	OR (95% CI)	P_h/I^2(%[illegible]
**Overall**	39 (18344/21711)	0.133 (0.087-0.203) *	0.000/75.3	1.777 (1.484-2.128) *	0.000/8[illegible]
**Ethnicity**					
Caucasian	28 (15918/19349)	0.119 (0.099-0.144)	0.460/0.2	1.764 (1.553-2.004) *	0.000/6[illegible]
Asian	5 (988/924)	0.064 (0.028-0.149)	0.770/0.0	2.921 (2.027-4.207)	0.132/4[illegible]
Mixed	5 (1293/1228)	0.208 (0.027-1.615) *	0.000/90.4	1.249 (0.449-3.473) *	0.000/9[illegible]
**Source of control**					
PB	24 (14543/17661)	0.155 (0.084-0.285) *	0.000/82.6	1.533 (1.190-1.974) *	0.000/9[illegible]
HB	15 (3801/4050)	0.129 (0.088-0.180)	0.397/4.9	2.121 (1.888-2.382)	0.038/4[illegible]
**Type of control**					
Healthy controls	25 (13729/16408)	0.114 (0.093-0.141)	0.278/14.1	1.694 (1.411-2.034) *	0.000/7[illegible]
Non-diabetic controls	12 (339/4333)	0.127 (0.087-0.185)	0.705/0.0	1.675 (1.508-1.860) *	0.000/7[illegible]
**Blinding and/or Quality control**					
YES	23 (5485/6650)	0.133 (0,098-0.180)	0.598/0.0	1.609 (1.348-1.920) *	0.000/6[illegible]
NO	16 (12859/15061)	0.136 (0.062-0.298) *	0.000/88.7	2.018 (1.439-2.829) *	0.000/9[illegible]
**HWE**					
Compliant	35 (17562/20898)	0.140 (0.090-0.218) *	0.000/76.0	1.785 (1.487-2.143) *	0.000/8[illegible]
In Violation/NR	4 (782/813)	0.065 (0.028-0.149)	0.782/0.0	1.359 (0.335-5.512) *	0.000/8[illegible]
**Sensitivity analysis**					
**Overall**	27 (6696/7887)	0.152 (0.117-0.196)	0.643/0.0	1.727 (1.487-2.007) *	0.000/6[illegible]
**Ethnicity**					
Caucasian	22 (6042/7098)	0.152 (0.116-0.199)	0.665/0.0	1.743 (1.503-2.022) *	0.000/6[illegible]
Asian	3 (387/342)	0.053 (0.010-0.273)	0.629/0.0	1.956 (1.174-3.257)	0.295/1[illegible]
**Geographic region**					
Europe	17 (4342/4725)	0.170 (0.128-0.225)	0.689/0.0	1.569 (1.303-1.891) *	0.000/7[illegible]
Asia	5 (643/713)	0.068 (0.016-0.287)	0.673/0.0	2.249 (1.480-3.657)	0.321/1[illegible]
**Source of control**					
PB	15 (3443/4450)	0.159 (0.112-0.227)	0.644/0.0	1.452 (1.165-1.811) *	0.000/6[illegible]
HB	12 (3253/3437)	0.140 (0.097-0.202)	0.442/0.0	2.035 (1.804-2.295)	0.349/9[illegible]
**Type of control**					
Healthy controls	17 (3782/4234)	0.156 (0.111-0.220)	0.406/4.2	1.671 (1.329-2.100) *	0.000/7[illegible]
Non-diabetic controls	10 (339/4333)	0.139 (0.092-0.210)	0.733/0.0	1.728 (1.540-1.938)	0.031/5[illegible]

**Egger's test**		
**P_E**	0.381	0.994

HB = hospital-based studies, PB = population-based studies, *=Random effects model.

Pooled analysis for PTPTN22 rs1310182

Table 4 illustrates the pooled analysis of the PTPN22 rs1310182 polymorphism and its association with T1D risk, based on six case-control studies involving 842 cases and 801 controls. No statistically significant associations were found in any models (CC vs. TT: OR = 0.560, 95% CI = 0.270-1.162; CT vs. CC: OR = 1.325, 95% CI = 0.740-2.371), with consistent results across subgroup analyses based on control source stratification.

Table 4 Meta-analysis of the association of PTPN22 rs1310182 polymorphism with risk of T1D.

Variable	n (Cases/Controls)	CC vs. TT		CT vs.CC	
		OR (95% CI)	P_h/I^2(%)	OR (95% CI)	P_h/I^2(%)
**Overall**	6 (842/801)	0.560 (0.270-1.162) *	0.000/80.3	1.325 (0.740-2.371) *	0.004/80.5
**Ethnicity**					
Caucasian	4 (519/451)	0.769 (0.331-1.785) *	0.005/76.5	1.306 (0.473-3.601) *	0.000/87.7
**Source of control**					
HB	4 (552/552)	0.465 (0.177-1.221) *	0.000/83.6	1.562 (0.635-3.844) *	0.000/86.0
**Blinding and/or Quality control**					
YES	5 (658/622)	0.583 (0.244-1.391) *	0.000/84.1	1.297 (0.609-2.762) *	0.000/83.7
**Sensitivity analysis**					
**Overall**	4 (559/522)	0.465 (0.177-1.221) *	0.000/83.6	1.562 (0.635-3.844) *	0.000/86.0
**Ethnicity**					
Caucasian	3 (420/351)	0.619 (0.207-1.854) *	0.006/80.4	1.687 (0.433-6.569) *	0.000/90.6
**Source of control**					
HB	4 (559/522)	0.465 (0.177-1.221) *	0.000/83.6	1.562 (0.635-3.844) *	0.000/86.0
**Egger's test**					
**P_E**		0.854		0.616	

HB=hospital-based studies, PB=population-based studies, *=Random effects model.

Heterogeneity and sensitivity analyses

Heterogeneity was observed for both SNPs in overall and multi-subgroup analyses. For rs2476601, significant heterogeneity was noted with the following I^2 and p-values: CC vs. TT (I^2 = 75.3%, $P<0.001$), CT vs. CC (I^2 = 87.1%, $P<0.001$), CC vs. CT+TT (I^2 = 89.7%, $P<0.001$), CT+CC vs. TT (I^2 = 74.3%, $P<0.001$), and C vs. T (I^2 = 95.5%, $P<0.001$), indicating significant heterogeneity across all five inheritance patterns. Similarly, for rs1310182, significant heterogeneity was observed: CC vs. TT (I^2 = 80.3%, $P<0.001$), CT vs. CC (I^2 = 80.5%, P = 0.004), CC vs. CT+TT (I^2 = 83.2%, $P<0.001$), CT+CC vs. TT (I^2 = 93.5%, $P<0.001$), and C vs. T (I^2 = 85.5%, $P<0.001$). We conducted meta-regression analyses to identify potential sources of heterogeneity, including ethnicity, sample size, control source, case source, HWE, family history, association assessment with appropriate statistics, and adjustment for confounders and quality scores. The results demonstrated that ethnicity (C vs. T: P = 0.016) and HWE (C vs. T: P = 0.024) were identified as the causes of the PTPN22 rs476601 polymorphism and T1D risk source of heterogeneity between studies.

To assess the stability of the included studies, sensitivity analyses were performed using a case-by-case exclusion method. For PTPN22 rs2476601, excluding the study by Chelala et al [31]. reduced heterogeneity from I^2 = 75.3% to 0.0% (P = 0.518). In contrast, excluding individual studies for PTPN22 rs1310182 did not significantly affect stability. We also compared study characteristics and conducted subgroup analyses based on ethnicity and

study quality to explain heterogeneity (**Fig 2**). In Caucasian and Asian populations, rs2476601 was negatively associated with T1D risk (CC vs. TT: Caucasian OR = 0.119, 95% CI = 0.099-0.144; Asian OR = 0.064, 95% CI = 0.028-0.149). However, in mixed-race populations, rs2476601 showed no association with T1D risk (CC vs. TT: OR = 0.208, 95% CI = 0.027-1.615). Subsequently, when only high-quality and HWE studies were included, the OR for the overall study and subgroup analyses remained largely unaffected.

Fig.2 Forest plot of subgroup analysis on PTPN22 rs2476601 and rs1310182 polymorphisms (CC vs.TT) by ethnicity and study quality. [A: Ethnicity (Caucasian, Asian, Mixed and Indian); B: Study quality (HWE and Quality score>12)].

Publication bias

In this study, publication bias was assessed using the Begg funnel plot and Egger's test. If any bias was detected, results were adjusted using the non-parametric trim and fill method. The funnel plots for all models (**Figure 4**) appeared approximately symmetric, and Egger's test results were all greater than 0.05 (rs2476601: CC vs. TT: P = 0.381; CT vs. CC: P = 0.994; CC vs. CT+TT: P = 0.544; CT+CC vs. TT: P = 0.352 and C vs. T: P = 0.875, **Table 3**; rs1310182: CC vs. TT: P = 0.854; CT vs. CC: P = 0.616; CC vs. CT+TT: P = 0.726; CT+CC vs. TT: P = 0.720 and C vs. T: P = 0.683, **Table 4**), indicating no evidence of publication bias.

Fig 3 Begg's funnel plot of PTPN22 polymorphisms and T1D susceptibility in the general population (CTvs.TT).

Credibility of the identified genetic associations

To assess the credibility of the study, we employed FPRP, BEDP, and Venice criteria, supporting a high-confidence association. The criteria included: (1) statistically significant associations were observed in at least two genetic models, i.e. P-values for the Z-test were < 0.05. (2) FPRP < 0.2 and BFDP < 0.8, (3) statistical power > 0.8 and (4) I^2 < 50%. The lower standard of "less credible certainty" is applied when the following conditions are met: (1) at least one genetic model had a P-value of 0.05.; (2) statistical power was between 50% and 79%, or FPRP > 0.2 or I^2 > 50%. **Table 5** presents the confidence assessment results for the PTPN22 rs2476601 polymorphism and its association with T1D susceptibility. The meta-analysis indicated that in Caucasian, the rs2476601 polymorphism was associated with T1D risk, yielding a "positive result with a high degree of confidence". Conversely, a "less credible positive result" was found in Asians, and no reliable positive results were found in population-based and hospital-based sources. Given the limited sample size, it is possible that the observed effect for rs1310182 may not be present or significant. Consequently, the results are subject to a high level of uncertainty, and the calculation of FPRP and BFDP values is not feasible.

Table 5 Credibility of the current meta-analysis.

Variables	Model	OR (95% CI)	I^2 (%)	Statistical power	Credibility Prior probability of 0.001	
					FPRP	BFDP
**PTPN22 rs2476601**						
**Overall**	CC vs.TT	0.133 (0.087-0.203)	75.3	0.004	0.590	< 0.001
	CT vs.CC	1.777 (1.484-2.128)	87.1	0.033	< 0.001	< 0.001
	CC vs. CT+TT	0.212 (0.172-0.262)	89.7	0.215	0.025	< 0.001
	CT+CC vs. TT	0.341 (0.231-0.502)	71.3	0.001	0.611	0.005
	C vs. T	0.547 (0.426-	95.5	0.059	0.031	0.084

		0.701)				
**Caucasian**	CC vs.TT	0.119 (0.099-0.144)	0.2	0.354	0.016	< 0.001
	CT vs.CC	1.764 (1.553-2.004)	68.6	0.954	0.006	< 0.001
	CC vs. CT+TT	0.242 (0.208-0.281)	75.6	0.782	0.007	< 0.001
	CT+CC vs. TT	0.295 (0.246-0.355)	0.0	0.402	0.014	0.994
	C vs. T	0.553 (0.429-0.714)	0.0	0.076	0.068	0.197
**Asian**	CC vs.TT	0.064 (0.028-0.149)	0.0	< 0.001	0.869	0.005
	CT vs.CC	2.921 (2.027-4.207)	43.4	< 0.001	0.047	0.001
	CC vs. CT+TT	0.074 (0.033-0.167)	66.7	< 0.001	0.857	0.005
	CT+CC vs. TT	0.306 (0.137-0.683)	0.0	0.029	0.993	0.991
	C vs. T	0.333 (0.245-0.454)	0.0	< 0.001	0.001	< 0.001
**PB**	CC vs.TT	0.155 (0.084-0.285)	82.6	< 0.001	0.597	0.002
	CT vs.CC	1.533 (1.190-1.974)	91.0	0.433	0.681	0.961
	CC vs. CT+TT	0.252 (0.193-0.328)	90.6	0.064	0.081	< 0.001
	CT+CC vs. TT	0.382 (0.215-0.679)	80.5	0.029	0.973	0.969
	C vs. T	0.724 (0.549-0.955)	94.6	0.720	0.969	0.997
**HB**	CC vs.TT	0.129 (0.088-0.180)	4.9	0.016	0.264	< 0.001
	CT vs.CC	2.121 (1.888-2.382)	43.3	0.989	0.006	< 0.001
	CC vs. CT+TT	0.157 (0.106-0.232)	88.3	0.006	0.478	< 0.001
	CT+CC vs. TT	0.309 (0.221-0.433)	0.0	< 0.001	0.002	< 0.001
	C vs. T	0.349 (0.213-0.572)	95.5	0.005	0.853	< 0.001
**HWE and Quality score > 12**						
**Overall**	CC vs.TT	0.152 (0.117-0.196)	0.0	0.079	0.067	< 0.001
	CT vs.CC	1.727 (1.487-2.007)	65.3	0.033	< 0.001	< 0.001

	CC vs. CT+TT	0.228 (0.188-0.278)	78.0	0.297	0.018	< 0.001
	CT+CC vs. TT	0.334 (0.262-0.426)	0.0	0.101	0.052	< 0.001
	C vs. T	0.546 (0.393-0.759)	95.1	0.117	0.730	0.901
**Caucasian**	CC vs.TT	0.152 (0.116-0.199)	0.0	0.056	0.091	< 0.001
	CT vs.CC	1.743 (1.503-2.022)	62.9	0.024	< 0.001	< 0.001
	CC vs. CT+TT	0.244 (0.203-0.294)	74.1	0.391	0.014	< 0.001
	CT+CC vs. TT	0.346 (0.267-0.448)	0.0	0.071	0.073	< 0.001
	C vs. T	0.562 (0.388-0.816)	95.9	0.185	0.930	0.981
**Asian**	CC vs.TT	0.053 (0.010-0.273)	0.0	0.001	0.997	0.994
	CT vs.CC	1.956 (1.174-3.257)	18.0	0.154	0.985	0.994
	CC vs. CT+TT	0.113 (0.033-0.387)	72.0	0.002	0.995	0.989
	CT+CC vs. TT	0.210 (0.045-0.969)	0.0	0.069	0.998	0.998
	C vs. T	0.420 (0.264-0.666)	0.0	0.025	0.901	0.888
**PB**	CC vs.TT	0.159 (0.112-0.227)	0.0	0.010	0.348	< 0.001
	CT vs.CC	1.452 (1.165-1.811)	69.7	0.614	0.604	0.963
	CC vs. CT+TT	0.273 (0.213-0.350)	73.0	0.090	0.059	< 0.001
	CT+CC vs. TT	0.386 (0.276-0.541)	10.6	0.001	0.041	0.003
**HB**	CC vs.TT	0.140 (0.097-0.202)	0.0	0.009	0.388	< 0.001
	CT vs.CC	2.035 (1.804-2.295)	9.9	0.981	0.006	< 0.001
	CC vs. CT+TT	0.181 (0.132-0.250)	82.5	0.019	0.230	< 0.001
	CT+CC vs. TT	0.302 (0.211-0.433)	0.0	< 0.001	0.009	< 0.001
	C vs. T	0.335 (0.191-0.588)	96.3	0.007	0.889	0.875

HB = hospital-based studies, PB = population-based studies, HWE= Hardy-Weinberg equilibrium.

Discussion

T1D represents a clinical form of diabetes mellitus characterized by the absolute insufficiency of insulin due to the destruction of pancreatic β-cells [1]. It is characterized by a proclivity towards ketosis and arises from a multifaceted interplay of genetic and environmental elements [66]. Extensive evidence underscores the varying susceptibility to T1D among diverse populations, with individual genetic factors emerging as pivotal determinants in the disease's pathogenesis [6]. Alongside the HLA region, the PTPN22 gene has now been acknowledged as a prominent genetic predisposition for autoimmunity [66]. Playing a critical role in immune regulation, the PTPN22 gene encodes a protein tyrosine phosphatase crucial in T cell receptor signaling and immune tolerance maintenance [67,68]. Notably, variations within the PTPN22 gene, notably the R620W polymorphism, have been linked to heightened risks of autoimmune disorders, including type 1 diabetes, by potentially disrupting normal immune function and fostering autoimmunity in those with T1D. Recent research underscores the substantial impact of the PTPN22 gene on T1D susceptibility. Despite this, a review of existing literature has revealed inconsistencies in earlier studies regarding the relationship between PTPN22 SNP and T1D susceptibility [13,14,34,35]. This investigation systematically scrutinized 43 articles to furnish evidence supporting the link between PTPN22 rs247660 and rs1310182 polymorphisms and the risk of T1D development.

In conclusion, the rs2476601 polymorphism significantly increased the risk of T1D, especially in Caucasians and Asians. Furthermore, in stratified analyses of subgroups, matched studies, unblinded and quality controlled, blinded and/or quality controlled, and HWE, an association between the rs2476601 polymorphism and T1D risk was again demonstrated. Given the significant between-group heterogeneity observed in the overall analysis, we performed subgroup analyses stratified by ethnicity as well as further sensitivity analyses, and the rs2476601 polymorphism was again found to be associated with T1D risk in the overall analysis as well as in Caucasians and Asians. Previous meta-analyses have suggested that the PTPN22 rs2476601 polymorphism may be a risk factor for T1D in Caucasians [7–10]. Our results are generally consistent with these studies and support the conclusion that this polymorphism increases susceptibility to T1D in Caucasians, suggesting that our study is reliable. Because several studies have addressed the PTPN22 rs1310182 polymorphism, we pooled data from several independent studies to assess the consistency and heterogeneity of the results. For the rs1310182 polymorphism, there were no significant results indicating an association between the rs1310182 polymorphism and T1D risk.

Given the considerable volume of genomic data being generated, a comprehensive correction was applied using the FPRP, BFDP, and Venice criteria. Our findings showed no correlation between the PTPN22 rs2476601 polymorphism and T1D risk across four genetic models, with $I^2 > 75\%$. However, correlations were observed in Caucasian and Asian subgroups based on ethnicity origin. The case source (including HB and PB) showed a low correlation with T1D risk. After correcting the sensitivity analysis results with the FPRP, BFDP, and Venice criteria, significant correlations appeared in the overall analysis, with a marked decline in significance for Asians. This indicates that integrating quantitative synthesized and low-quality data may affect result reliability. The rs2476601 polymorphism is considered a "high confidence positive result" for T1D susceptibility. In contrast, results for Asians, HB, and PB sources are "false positives" with low confidence, and other results are unreliable. Further high-quality studies are needed for more definitive conclusions.

A comparison of the characteristics of this study with those of previous meta-analyses is provided in **Supplementary Table 2**. This meta-analysis includes 45 studies, significantly expanding on earlier work. Of these, 39 studies examined the PTPN22 rs2476601 polymorphism, and 6 focused on the rs1310182 polymorphism. This study used a larger sample size than previous analyses, incorporating the most recent studies from 2023, whereas earlier studies were mainly published before 2013. Review of past studies, Bottini first studied the PTPN22 gene polymorphism and T1D in 2002 [69], and a follow-up in 2004 showed an increased T1D risk in North America and Sardinia [6]. Subsequently, a 2006 meta-analysis by Lee linked the

PTPN22 SNP to autoimmune diseases like rheumatoid arthritis, systemic lupus erythematosus, Graves' disease, T1D, and juvenile idiopathic arthritis [70]. Moreover, several later analyses suggested that the PTPN22 rs2476601 polymorphism may be a T1D risk factor in Caucasians, which aligns with our findings [7–10]. However, previous meta-analyses had significant flaws. For instance, only two studies stratified analyses by ethnicity, revealing inconsistencies across studies from various countries [7,10]. Additionally, data overlap, inclusion of unnecessary data, and omission of study quality assessments likely skewed results by incorporating low-quality studies [10]. Furthermore, one analyse ignored Hardy-Weinberg equilibrium, risking selection bias or genotyping errors [10]. All lacked sensitivity assessments and had incomplete study searches. Different genetic models were used, with some analyses comparing three models [7,9,10] and others four [8], which potentially caused false negatives. Importantly, previous meta-analyses failed to assess the probability of false-positive results, raising credibility concerns. Notably, Wakefeld in 2007 proposed a more accurate Bayesian approach to determine genetic error detection in epidemiologic surveys. Many factors can contribute to error and bias, such as genotyping error and phenotypic misclassification, of which statistical power is an important factor. A large body of evidence (statistical power >80%) can achieve high statistical significance or reduce false discovery rates [71]. By neglecting result confidence, previous studies may have led to unreliable conclusions.

In order to gain further insight into the relationship between the PTPN22 polymorphism and the risk of developing T1D, we conducted an updated meta-analysis. In this analysis, we employed a larger sample size and conducted quality assessments and Hardy-Weinberg equilibrium tests on the relevant studies. Additionally, meta-regression analyses were conducted based on nine factors: ethnicity, sample size, control source, case source, HWE, family history, association assessment with appropriate statistics, adjustment for confounders, and quality scores. The objective was to identify sources of heterogeneity. Furthermore, stratified analyses were conducted based on the studies' epidemiological characteristics, with particular attention paid to sensitivity analyses. These involved the selection of high-quality studies with high composite scores and conformity to HWE, with the aim of avoiding random errors and confounding bias. Finally, significant results were corrected using the FPRP test, BFDP test, and Venice criteria.

Although a number of strategies were used to mitigate the problems of previous studies, this study still has some limitations. First, only published articles were searched, so the omission of some results may be unavoidable. Second, the paucity of studies with partial subgroup analyses and the lack of relevant data from African and Indian populations prevented us from assessing the impact of these polymorphisms on the pathogenesis of T1D in these populations, highlighting the need for broader multi-ethnic investigations. Third, studies varied in their selection of controls - some controls explicitly excluded T1D, while others were asymptomatic - which may have introduced misclassification bias by not fully excluding potential diabetes cases. Fourth, the analyses did not control for potential confounders, including age, sex, season, dietary variables, and study design. Therefore, more precise analyses should be done in the future when sufficient data are available.

Supplemental Table 2 Included studies of PTPN22 polymorphisms in T1D within the meta-analyses.

Conclusion

A comprehensive analysis of 43 articles concluded that the PTPN22 rs2476601 polymorphism increases susceptibility to T1D in Caucasian populations, suggesting a positive association with T1D. However, no definitive conclusion could be drawn regarding the relationship between PTPN22 rs1310182 and T1D susceptibility. Further studies across diverse populations are necessary to confirm these associations. Additionally, investigating the biological significance of functional SNPs in T1D activity and expression is essential for understanding their impact on the disease.

Supporting Information

S1 Table. Scale for quality assessment of molecular association studies of T1D
(DOCX)
S2 Table. Included studies of PTPN22 polymorphisms in T1D within the meta-analyses.
(DOCX)
S3 Text. PRISMA 2020 checklist.
(DOC)
S4 Text. plosone-checklist.
(PDF)

Acknowledgments
We gratefully acknowledge the contributors to all the original studies included in this meta-analysis.

Funding
The authors received no specific funding for this work.

Conflict of interest
There are no conflicts of interest among the authors.

Conflict of interest
The authors declare that the research was conducted in the absence of any commercial or financial relationships that could be construed as a potential conflict of interest.

Author contributions
Conceptualization: Yu Su.
Data curation: Yu Su, Xue Li, Pei-dong Wu, Peng-fei Fang.
Formal analysis: Xiao-feng He.
Methodology: Xiao-feng He.
Supervision: Fei-fei Wu.
Validation: Yu Su, Yu-long Zhang.
Writing – original draft: Yu Su, Xue Li, Pei-dong Wu.
Writing – review & editing: Yu Su, Xue Li, Pei-dong Wu.

Reference

1. DiMeglio LA, Evans-Molina C, Oram RA. Type 1 diabetes. Lancet. 2018;391: 2449–2462. doi:10.1016/S0140-6736(18)31320-5
2. Stanford SM, Bottini N. PTPN22: the archetypal non-HLA autoimmunity gene. Nat Rev Rheumatol. 2014;10: 602–611. doi:10.1038/nrrheum.2014.109
3. Jassim BA, Lin J, Zhang Z-Y. PTPN22: structure, function, and developments in inhibitor discovery with applications for immunotherapy. Expert Opin Drug Discov. 2022;17: 825–837. doi:10.1080/17460441.2022.2084607
4. N B, Ej P. Tyrosine phosphatase PTPN22: multifunctional regulator of immune signaling, development, and disease. Annual review of immunology. 2014;32. doi:10.1146/annurev-immunol-032713-120249
5. Dai X, James RG, Habib T, Singh S, Jackson S, Khim S, et al. A disease-associated PTPN22 variant promotes systemic autoimmunity in murine models. J Clin Invest. 2013;123: 2024–2036. doi:10.1172/JCI66963

6. Bottini N, Musumeci L, Alonso A, Rahmouni S, Nika K, Rostamkhani M, et al. A functional variant of lymphoid tyrosine phosphatase is associated with type I diabetes. Nat Genet. 2004;36: 337–338. doi:10.1038/ng1323
7. Peng H, Zhou M, Xu W-D, Xu K, Zhai Y, Li R, et al. Association of PTPN22 C1858T polymorphism and type 1 diabetes: a meta-analysis. Immunol Invest. 2012;41: 484–496. doi:10.3109/08820139.2012.664226
8. Tang S, Peng W, Wang C, Tang H, Zhang Q. Association of the PTPN22 gene (+1858C/T, -1123G/C) polymorphisms with type 1 diabetes mellitus: a systematic review and meta-analysis. Diabetes Res Clin Pract. 2012;97: 446–452. doi:10.1016/j.diabres.2012.04.011
9. Wang X-F, Chen Z-X, Shao Y-C, Ma Y-S, Zhang F, Zhang L, et al. Population-based and family-based studies on the protein tyrosine phosphatase non-receptor 22 gene polymorphism and type 1 diabetes: a meta-analysis. Gene. 2013;517: 191–196. doi:10.1016/j.gene.2012.12.076
10. Young Ho, Song GG. Meta-analysis of the family-based association between the PTPN22 C1858T polymorphism and type 1 diabetes. Mol Biol Rep. 2013;40: 211–215. doi:10.1007/s11033-012-2051-8
11. Bottini N, Vang T, Cucca F, Mustelin T. Role of PTPN22 in type 1 diabetes and other autoimmune diseases. Semin Immunol. 2006;18: 207–213. doi:10.1016/j.smim.2006.03.008
12. Kawasaki E, Awata T, Ikegami H, Kobayashi T, Maruyama T, Nakanishi K, et al. Systematic search for single nucleotide polymorphisms in a lymphoid tyrosine phosphatase gene (PTPN22): association between a promoter polymorphism and type 1 diabetes in Asian populations. Am J Med Genet A. 2006;140: 586–593. doi:10.1002/ajmg.a.31124
13. Heneberg P, Kocková L, Čecháková M, Daňková P, Černá M. Autoimmunity-Associated PTPN22 Polymorphisms in Latent Autoimmune Diabetes of the Adult Differ from Those of Type 1 Diabetes Patients. Int Arch Allergy Immunol. 2018;177: 57–68. doi:10.1159/000489225
14. Taniyama M, Maruyama T, Tozaki T, Nakano Y, Ban Y. Association of PTPN22 haplotypes with type 1 diabetes in the Japanese population. Hum Immunol. 2010;71: 795–798. doi:10.1016/j.humimm.2010.05.016
15. Brzoza Z, Grzeszczak W, Rogala B, Trautsolt W, Moczulski D. PTPN22 polymorphism presumably plays a role in the genetic background of chronic spontaneous autoreactive urticaria. Dermatology. 2012;224: 340–345. doi:10.1159/000339332
16. Carlton VEH, Hu X, Chokkalingam AP, Schrodi SJ, Brandon R, Alexander HC, et al. PTPN22 genetic variation: evidence for multiple variants associated with rheumatoid arthritis. Am J Hum Genet. 2005;77: 567–581. doi:10.1086/468189
17. Zoledziewska M, Perra C, Orrù V, Moi L, Frongia P, Congia M, et al. Further evidence of a primary, causal association of the PTPN22 620W variant with type 1 diabetes. Diabetes. 2008;57: 229–234. doi:10.2337/db07-0289
18. Žak R, Navasardyan L, Hunák J, Martinů J, Heneberg P. PTPN22 intron polymorphism rs1310182 (c.2054-852T>C) is associated with type 1 diabetes mellitus in patients of Armenian descent. PLoS One. 2023;18: e0286743. doi:10.1371/journal.pone.0286743
19. Abbasi F, Soltani S, Saghazadeh A, Soltaninejad E, Rezaei A, Zare Bidoki A, et al. PTPN22 Single-Nucleotide Polymorphisms in Iranian Patients with Type 1 Diabetes Mellitus. Immunol Invest. 2017;46: 409–418. doi:10.1080/08820139.2017.1288239
20. Zouidi F, Stayoussef M, Bouzid D, Fourati H, Abida O, Ayed MB, et al. Contribution of PTPN22, CD28, CTLA-4 and ZAP-70 variants to the risk of type 1 diabetes in Tunisians. Gene. 2014;533: 420–426. doi:10.1016/j.gene.2013.09.112
21. Sharma C, R Ali B, Osman W, Afandi B, Aburawi EH, Beshyah SA, et al. Association of variants in PTPN22, CTLA-4, IL2-RA, and INS genes with type 1 diabetes in Emiratis. Ann Hum Genet. 2021;85: 48–57. doi:10.1111/ahg.12406
22. Mj P, Je M, Pm B, I B, Tc H, Cd M, et al. The PRISMA 2020 statement: an updated guideline for reporting systematic reviews. BMJ (Clinical research ed). 2021;372. doi:10.1136/bmj.n71

23. Smyth D, Cooper JD, Collins JE, Heward JM, Franklyn JA, Howson JMM, et al. Replication of an association between the lymphoid tyrosine phosphatase locus (LYP/PTPN22) with type 1 diabetes, and evidence for its role as a general autoimmunity locus. Diabetes. 2004;53: 3020–3023. doi:10.2337/diabetes.53.11.3020
24. Zheng W, She J-X. Genetic association between a lymphoid tyrosine phosphatase (PTPN22) and type 1 diabetes. Diabetes. 2005;54: 906–908. doi:10.2337/diabetes.54.3.906
25. Kahles H, Ramos-Lopez E, Lange B, Zwermann O, Reincke M, Badenhoop K. Sex-specific association of PTPN22 1858T with type 1 diabetes but not with Hashimoto's thyroiditis or Addison's disease in the German population. Eur J Endocrinol. 2005;153: 895–899. doi:10.1530/eje.1.02035
26. Gomez LM, Anaya J-M, Gonzalez CI, Pineda-Tamayo R, Otero W, Arango A, et al. PTPN22 C1858T polymorphism in Colombian patients with autoimmune diseases. Genes Immun. 2005;6: 628–631. doi:10.1038/sj.gene.6364261
27. Zhernakova A, Eerligh P, Wijmenga C, Barrera P, Roep BO, Koeleman BPC. Differential association of the PTPN22 coding variant with autoimmune diseases in a Dutch population. Genes Immun. 2005;6: 459–461. doi:10.1038/sj.gene.6364220
28. Hermann R, Lipponen K, Kiviniemi M, Kakko T, Veijola R, Simell O, et al. Lymphoid tyrosine phosphatase (LYP/PTPN22) Arg620Trp variant regulates insulin autoimmunity and progression to type 1 diabetes. Diabetologia. 2006;49: 1198–1208. doi:10.1007/s00125-006-0225-4
29. Fedetz M, Matesanz F, Caro-Maldonado A, Smirnov II, Chvorostinka VN, Moiseenko TA, et al. The 1858T PTPN22 gene variant contributes to a genetic risk of type 1 diabetes in a Ukrainian population. Tissue Antigens. 2006;67: 430–433. doi:10.1111/j.1399-0039.2006.00591.x
30. Steck AK, Liu S-Y, McFann K, Barriga KJ, Babu SR, Eisenbarth GS, et al. Association of the PTPN22/LYP gene with type 1 diabetes. Pediatr Diabetes. 2006;7: 274–278. doi:10.1111/j.1399-5448.2006.00202.x
31. Chelala C, Duchatelet S, Joffret M-L, Bergholdt R, Dubois-Laforgue D, Ghandil P, et al. PTPN22 R620W functional variant in type 1 diabetes and autoimmunity related traits. Diabetes. 2007;56: 522–526. doi:10.2337/db06-0942
32. Santiago JL, Martínez A, de la Calle H, Fernández-Arquero M, Figueredo MA, de la Concha EG, et al. Susceptibility to type 1 diabetes conferred by the PTPN22 C1858T polymorphism in the Spanish population. BMC Med Genet. 2007;8: 54. doi:10.1186/1471-2350-8-54
33. Nielsen C, Hansen D, Husby S, Lillevang ST. Sex-specific association of the human PTPN22 1858T-allele with type 1 diabetes. Int J Immunogenet. 2007;34: 469–473. doi:10.1111/j.1744-313X.2007.00720.x
34. Cinek O, Hradsky O, Ahmedov G, Slavcev A, Kolouskova S, Kulich M, et al. No independent role of the -1123 G>C and+2740 A>G variants in the association of PTPN22 with type 1 diabetes and juvenile idiopathic arthritis in two Caucasian populations. Diabetes Res Clin Pract. 2007;76: 297–303. doi:10.1016/j.diabres.2006.09.009
35. Baniasadi V, Das SN. No evidence for association of PTPN22 R620W functional variant C1858T with type 1 diabetes in Asian Indians. J Cell Mol Med. 2008;12: 1061–1062. doi:10.1111/j.1582-4934.2008.00222.x
36. Douroudis K, Prans E, Haller K, Nemvalts V, Rajasalu T, Tillmann V, et al. Protein tyrosine phosphatase non-receptor type 22 gene variants at position 1858 are associated with type 1 and type 2 diabetes in Estonian population. Tissue Antigens. 2008;72: 425–430. doi:10.1111/j.1399-0039.2008.01115.x
37. Dultz G, Matheis N, Dittmar M, Röhrig B, Bender K, Kahaly GJ. The protein tyrosine phosphatase non-receptor type 22 C1858T polymorphism is a joint susceptibility locus for immunthyroiditis and autoimmune diabetes. Thyroid. 2009;19: 143–148. doi:10.1089/thy.2008.0301
38. Smyth DJ, Cooper JD, Howson JMM, Walker NM, Plagnol V, Stevens H, et al. PTPN22 Trp620 explains the association of chromosome 1p13 with type 1 diabetes and shows a statistical interaction with HLA class II genotypes. Diabetes. 2008;57: 1730–1737. doi:10.2337/db07-1131

39. Korolija M, Renar IP, Hadzija M, Medvidović EP, Pavković P, Jokić M, et al. Association of PTPN22 C1858T and CTLA-4 A49G polymorphisms with Type 1 Diabetes in Croatians. Diabetes Res Clin Pract. 2009;86: e54-57. doi:10.1016/j.diabres.2009.09.012
40. Lavrikova EI, Nikitin AG, Seregin IA, Zil'berman LI, Tsitlidze NM, Kuraeva TL, et al. [Association of the C1858T polymorphism of the PTPN22 gene with type 1 diabetes]. Mol Biol (Mosk). 2009;43: 1040–1043.
41. Fichna M, Zurawek M, Januszkiewicz-Lewandowska D, Fichna P, Nowak J. PTPN22, PDCD1 and CYP27B1 polymorphisms and susceptibility to type 1 diabetes in Polish patients. Int J Immunogenet. 2010;37: 367–372. doi:10.1111/j.1744-313X.2010.00935.x
42. Kordonouri O, Hartmann R, Badenhoop K, Kahles H, Ilonen J. PTPN22 1858T allele is associated with younger age at onset of type 1 diabetes and is not related to subsequent thyroid autoimmunity. Hum Immunol. 2010;71: 731–732. doi:10.1016/j.humimm.2010.04.002
43. Chagastelles PC, Romitti M, Trein MR, Bandinelli E, Tschiedel B, Nardi NB. Association between the 1858T allele of the protein tyrosine phosphatase nonreceptor type 22 and type 1 diabetes in a Brazilian population. Tissue Antigens. 2010;76: 144–148. doi:10.1111/j.1399-0039.2010.01480.x
44. Zhebrun D, Kudryashova Y, Babenko A, Maslyansky A, Kunitskaya N, Popcova D, et al. Association of PTPN22 1858T/T genotype with type 1 diabetes, Graves' disease but not with rheumatoid arthritis in Russian population. Aging (Albany NY). 2011;3: 368–373. doi:10.18632/aging.100305
45. Liu F, Liu J, Zheng T, Li Q, Wang C, Pan X, et al. The -1123G>C variant of PTPN22 gene promoter is associated with latent autoimmune diabetes in adult Chinese Hans. Cell Biochem Biophys. 2012;62: 273–279. doi:10.1007/s12013-011-9291-4
46. Kisand K, Uibo R. LADA and T1D in Estonian population — Two different genetic risk profiles. Gene. 2012;497: 285–291. doi:10.1016/j.gene.2012.01.089
47. Giza S, Goulas A, Gbandi E, Effraimidou S, Papadopoulou-Alataki E, Eboriadou M, et al. The role of PTPN22 C1858T gene polymorphism in diabetes mellitus type 1: first evaluation in Greek children and adolescents. Biomed Res Int. 2013;2013: 721604. doi:10.1155/2013/721604
48. Hadžija M, Korolija M, Vukadinović G, Hadžija M. Association of TNF-α and PTPN22 SNPs with the risk and clinical outcome of type 1 diabetes. Open Life Sciences. 2013;8: 513–519. doi:10.2478/s11535-013-0166-5
49. Almasi S, Aliparasti MR, Yazdchi-Marandi L, Aliasgarzadeh A, Sioofy-Khojine A, Mesri A, et al. Analysis of PTPN22 C1858T gene polymorphism in cases with type 1 diabetes of Azerbaijan, Northwest Iran. Cell Immunol. 2014;292: 14–18. doi:10.1016/j.cellimm.2014.08.007
50. Kumar N, Kaur G, Kanga U, Tandon N, Caillat-Zucman S, Mehra NK. Association of PTPN22+1858C/T polymorphism with Type 1 diabetes in the North Indian population. Int J Immunogenet. 2014;41: 318–323. doi:10.1111/iji.12129
51. Min R. Experimental study of T cells and related cytokines in autoimmune diabetes. Master, Peking Union Medical College. 2014.
52. Liu HW, Xu RY, Sun RP, Wang Q, Liu JL, Ge W, et al. Association of PTPN22 gene polymorphism with type 1 diabetes mellitus in Chinese children and adolescents. Genet Mol Res. 2015;14: 63–68. doi:10.4238/2015.January.15.8
53. Pawłowicz M, Filipów R, Krzykowski G, Stanisławska-Sachadyn A, Morzuch L, Kulczycka J, et al. Coincidence of PTPN22 c.1858CC and FCRL3 -169CC genotypes as a biomarker of preserved residual β-cell function in children with type 1 diabetes. Pediatr Diabetes. 2017;18: 696–705. doi:10.1111/pedi.12429
54. Alswat KA, Nasr A, Al Dubayee MS, Talaat IM, Alsulaimani AA, Mohamed IAA, et al. The Potential Role of PTPN-22 C1858T Gene Polymorphism in the Pathogenesis of Type 1 Diabetes in Saudi Population. Immunol Invest. 2018;47: 521–533. doi:10.1080/08820139.2018.1458109

55. El Fotoh WMMA, El Razek Midan DA, El Shalakany AH. Role of C1858T Polymorphism of Lymphoid Tyrosine Phosphatase in Egyptian Children and Adolescents with Type 1 Diabetes. Curr Diabetes Rev. 2019;16: 73–79. doi:10.2174/1573399814666180709102533
56. Rochmah N, Arief F, Faizi M, Basuki S. The association between PTPN22 C1858T gene polymorphism and type 1 diabetes mellitus: an Indonesian study. Annals of Medicine. 2023;55: 1211–1215. doi:10.1080/07853890.2023.2190162
57. Sharma C, R Ali B, Osman W, Afandi B, Aburawi EH, Beshyah SA, et al. Association of variants in PTPN22, CTLA-4, IL2-RA, and INS genes with type 1 diabetes in Emiratis. Ann Hum Genet. 2021;85: 48–57. doi:10.1111/ahg.12406
58. Žak R, Navasardyan L, Hunák J, Martinů J, Heneberg P. PTPN22 intron polymorphism rs1310182 (c.2054-852T>C) is associated with type 1 diabetes mellitus in patients of Armenian descent. PLoS One. 2023;18: e0286743. doi:10.1371/journal.pone.0286743
59. Mantel N, Haenszel W. Statistical aspects of the analysis of data from retrospective studies of disease. J Natl Cancer Inst. 1959;22: 719–748.
60. DerSimonian R, Laird N. Meta-analysis in clinical trials revisited. Contemp Clin Trials. 2015;45: 139–145. doi:10.1016/j.cct.2015.09.002
61. Begg CB, Mazumdar M. Operating characteristics of a rank correlation test for publication bias. Biometrics. 1994;50: 1088–1101.
62. Egger M, Davey Smith G, Schneider M, Minder C. Bias in meta-analysis detected by a simple, graphical test. BMJ. 1997;315: 629–634. doi:10.1136/bmj.315.7109.629
63. S D, R T. Trim and fill: A simple funnel-plot-based method of testing and adjusting for publication bias in meta-analysis. Biometrics. 2000;56. doi:10.1111/j.0006-341x.2000.00455.x
64. Wacholder S, Chanock S, Garcia-Closas M, El Ghormli L, Rothman N. Assessing the probability that a positive report is false: an approach for molecular epidemiology studies. J Natl Cancer Inst. 2004;96: 434–442. doi:10.1093/jnci/djh075
65. Jp I, P B, J L, Tr O, Ag U, P V, et al. Assessment of cumulative evidence on genetic associations: interim guidelines. International journal of epidemiology. 2008;37. doi:10.1093/ije/dym159
66. Knip M, Veijola R, Virtanen SM, Hyöty H, Vaarala O, Akerblom HK. Environmental triggers and determinants of type 1 diabetes. Diabetes. 2005;54 Suppl 2: S125-136. doi:10.2337/diabetes.54.suppl_2.s125
67. Cohen S, Dadi H, Shaoul E, Sharfe N, Roifman CM. Cloning and characterization of a lymphoid-specific, inducible human protein tyrosine phosphatase, Lyp. Blood. 1999;93: 2013–2024.
68. Gregersen PK, Behrens TW. Genetics of autoimmune diseases--disorders of immune homeostasis. Nat Rev Genet. 2006;7: 917–928. doi:10.1038/nrg1944
69. Bottini N, Bottini E, Gloria-Bottini F, Mustelin T. Low-molecular-weight protein tyrosine phosphatase and human disease: in search of biochemical mechanisms. Arch Immunol Ther Exp (Warsz). 2002;50: 95–104.
70. Lee YH, Rho YH, Choi SJ, Ji JD, Song GG, Nath SK, et al. The PTPN22 C1858T functional polymorphism and autoimmune diseases--a meta-analysis. Rheumatology (Oxford). 2007;46: 49–56. doi:10.1093/rheumatology/kel170
71. Wakefield J. A Bayesian Measure of the Probability of False Discovery in Genetic Epidemiology Studies. The American Journal of Human Genetics. 2007;81: 208–227. doi:10.1086/519024

Click here to access/download;Figure;Fig.1.docx

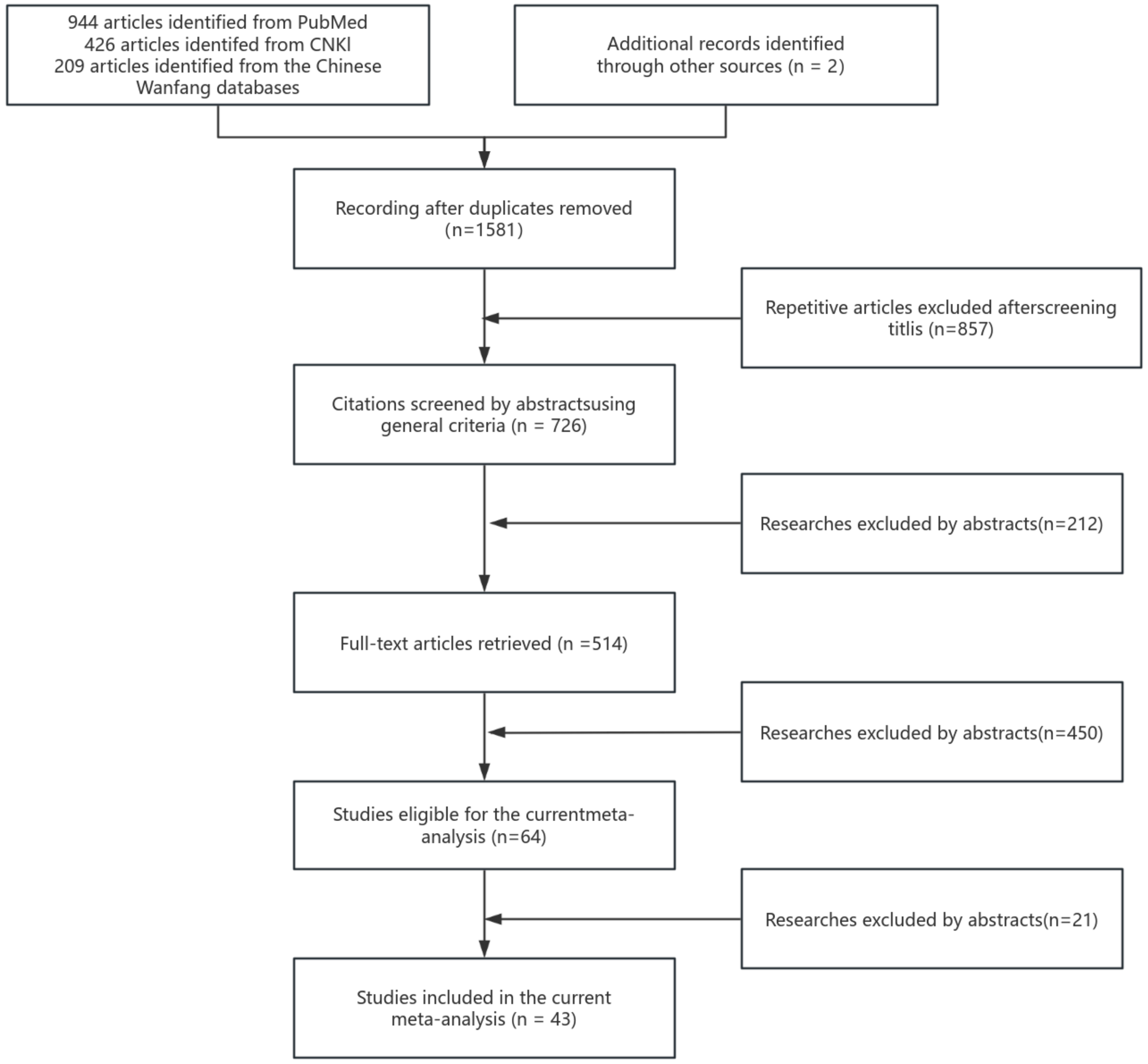

Fig. 1 Flowchart of Literature Retrieval Selection

Figure 2

Click here to access/download;Figure;Fig.2.docx

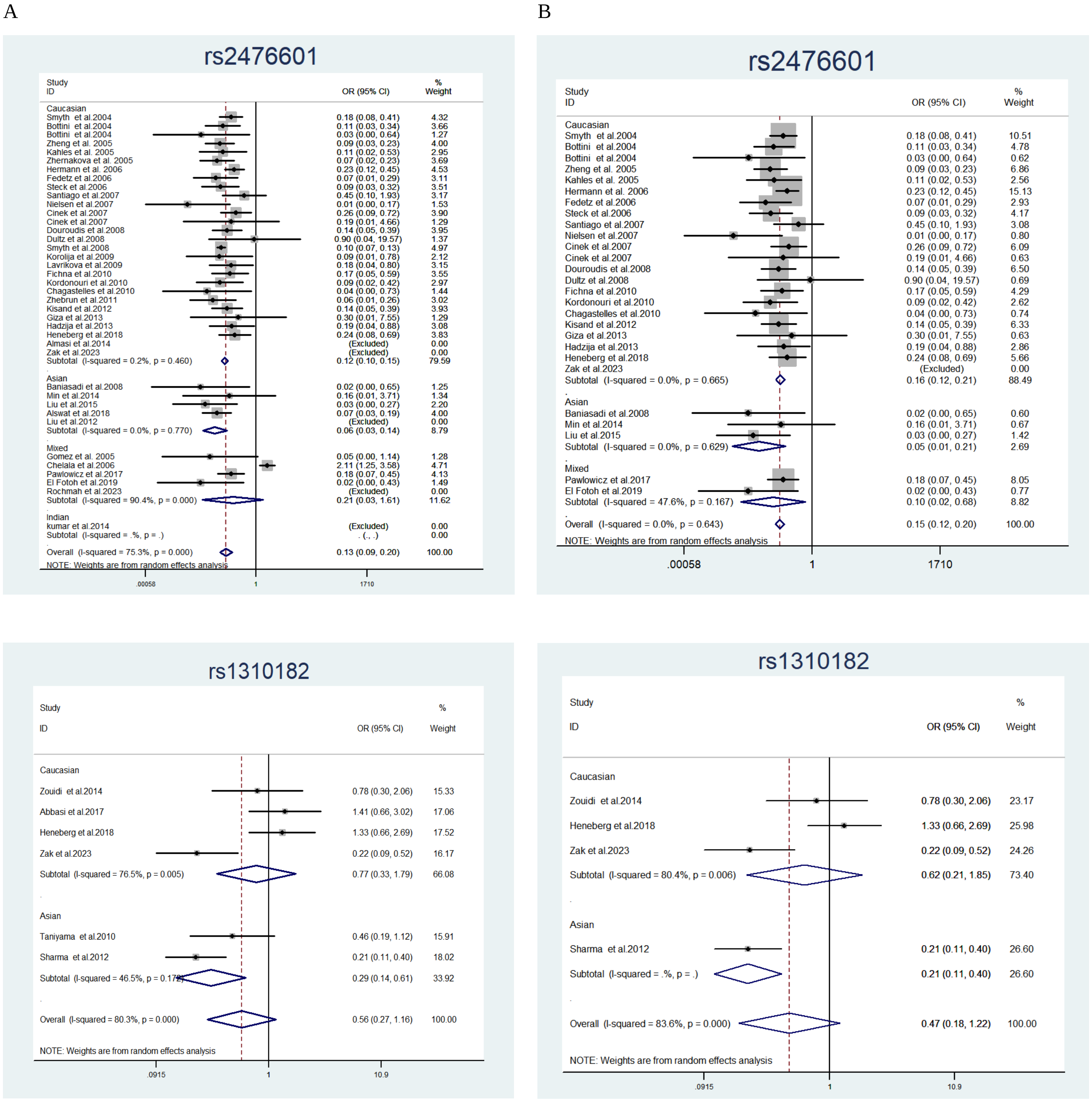

Fig.2 Forest plot of subgroup analysis on PTPN22 rs2476601 and rs1310182 polymorphisms (CC vs.TT) by ethnicity and study quality. (A): PTPN22 rs2476601 Ethnicity (Caucasian, Asian, Mixed and Indian); (B): Study quality (HWE and Quality score>12).

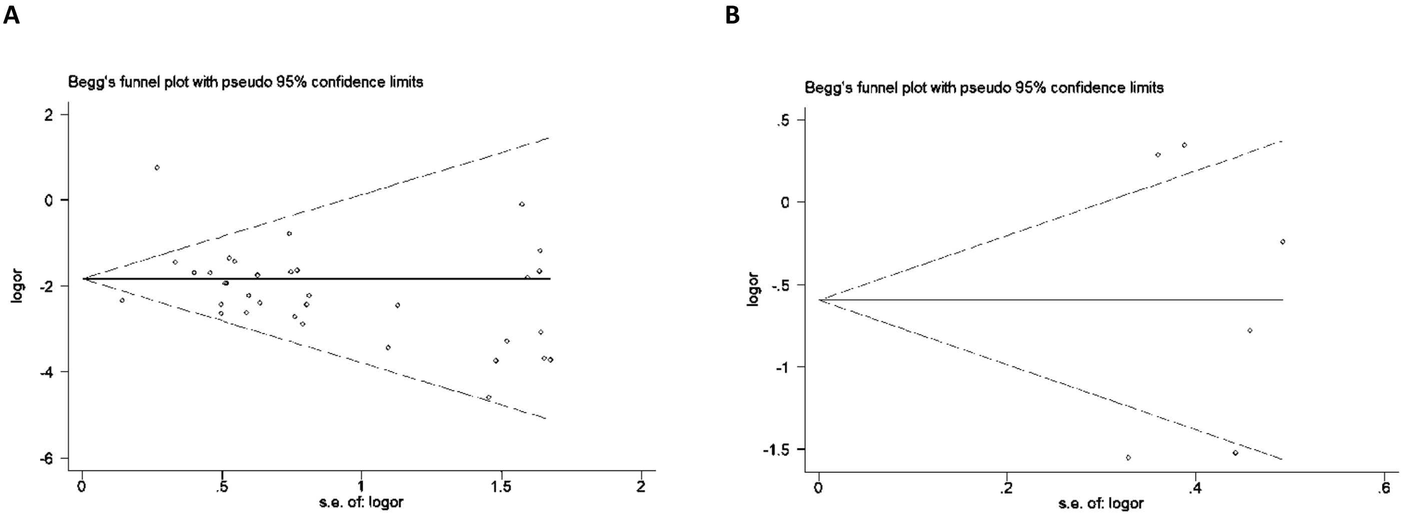

Fig 3 Begg's funnel plot to assess publication bias on the combined effects of PTPN22 polymorphisms with T1D risk in overall population(dominant model). [**A:** PTPN22（rs2476601); **B:** PTPN22（rs1310182)]

Click here to access/download
Supporting Information
Supplemental Table 1..docx

Click here to access/download
Supporting Information
Supplemental Table 2..docx

Click here to access/download
Supporting Information
Supplemental Text 3..doc

Click here to access/download
Supporting Information
Supplemental Text 4..pdf
