# Supplementary material for: The Association between PTPN22 SNPs and susceptibility to type 1 diabetes: An updated meta-analysis
Source: PLoS One. 2025 Apr 16;20(4):e0321624. doi: 10.1371/journal.pone.0321624 (PMC12002458; doi:10.1371/journal.pone.0321624)
Supplement: Attachment — Submitted filename: Response to Editor.docx [file pone.0321624.s010.docx]

Dear PLOS ONE Staff,

On behalf of all co-authors, I sincerely appreciate your constructive feedback and the opportunity to revise our manuscript titled "The Association between PTPN22 SNPs and Susceptibility to Type 1 Diabetes: An updated meta-analysis" (manuscript number: PONE-D-24-50318). We have carefully addressed each point raised in your review and detailed our responses below. Revised sections are marked in the manuscript, and supplementary files have been updated accordingly.

1.Please ensure that your manuscript meets PLOS ONE's style requirements, including those for file naming. The PLOS ONE style templates can be found at

https://journals.plos.org/plosone/s/file?id=wjVg/PLOSOne_formatting_sample_main_body.pdf and https://journals.plos.org/plosone/s/file?id=ba62/PLOSOne_formatting_sample_title_authors_affiliations.pdf

***Response:*** Thank you for providing the Author Formatting Checklist to ensure that our paper meets PLOS ONE's typesetting requirements for References, Tables, and Figures. We will carefully review the checklist and make the necessary adjustments to our submission files accordingly.

2.We note that your Data Availability Statement is currently as follows: [We gratefully acknowledge the contributors to all the original studies included in this meta-analysis.]

Please confirm at this time whether or not your submission contains all raw data required to replicate the results of your study. Authors must share the “minimal data set” for their submission. PLOS defines the minimal data set to consist of the data required to replicate all study findings reported in the article, as well as related metadata and methods (https://journals.plos.org/plosone/s/data-availability#loc-minimal-data-set-definition).

For example, authors should submit the following data:

- The values behind the means, standard deviations and other measures reported;

- The values used to build graphs;

- The points extracted from images for analysis.

Authors do not need to submit their entire data set if only a portion of the data was used in the reported study.

If your submission does not contain these data, please either upload them as Supporting Information files or deposit them to a stable, public repository and provide us with the relevant URLs, DOIs, or accession numbers. For a list of recommended repositories, please see https://journals.plos.org/plosone/s/recommended-repositories.

If there are ethical or legal restrictions on sharing a de-identified data set, please explain them in detail (e.g., data contain potentially sensitive information, data are owned by a third-party organization, etc.) and who has imposed them (e.g., an ethics committee). Please also provide contact information for a data access committee, ethics committee, or other institutional body to which data requests may be sent. If data are owned by a third party, please indicate how others may request data access.

***Response:*** We confirm that our submission includes all raw data required to replicate the results of our study. The data has been provided in the supplementary materials as per the journal’s requirements(Supporting Information files).

3.PLOS requires an ORCID iD for the corresponding author in Editorial Manager on papers submitted after December 6th, 2016. Please ensure that you have an ORCID iD and that it is validated in Editorial Manager. To do this, go to ‘Update my Information’ (in the upper left-hand corner of the main menu), and click on the Fetch/Validate link next to the ORCID field. This will take you to the ORCID site and allow you to create a new iD or authenticate a pre-existing iD in Editorial Manager.

***Response:*** The ORCID iD of the corresponding author (Fei-fei Wu: 0009-0000-0333-9833) has been added to the title page and verified in Editorial Manager.

4.Please include a new copy of Tables in your manuscript; the current table is difficult to read. Please follow the link for more information: https://blogs.plos.org/plos/2019/06/looking-good-tips-for-creating-your-plos-figures-graphics/

***Response:*** All tables (Tables 1-5) have been reformatted using Arial font for clarity. Headings have been bolded for better differentiation and improved readability. The updated tables are included in the manuscript and in the supplementary document.

5.As required by our policy on Data Availability, please ensure your manuscript or supplementary information includes the following:

A numbered table of all studies identified in the literature search, including those that were excluded from the analyses.

For every excluded study, the table should list the reason(s) for exclusion.

If any of the included studies are unpublished, include a link (URL) to the primary source or detailed information about how the content can be accessed.

A table of all data extracted from the primary research sources for the systematic review and/or meta-analysis. The table must include the following information for each study:

Name of data extractors and date of data extraction

Confirmation that the study was eligible to be included in the review.

All data extracted from each study for the reported systematic review and/or meta-analysis that would be needed to replicate your analyses.

If data or supporting information were obtained from another source (e.g. correspondence with the author of the original research article), please provide the source of data and dates on which the data/information were obtained by your research group.

If applicable for your analysis, a table showing the completed risk of bias and quality/certainty assessments for each study or outcome. Please ensure this is provided for each domain or parameter assessed. For example, if you used the Cochrane risk-of-bias tool for randomized trials, provide answers to each of the signalling questions for each study. If you used GRADE to assess certainty of evidence, provide judgements about each of the quality of evidence factor. This should be provided for each outcome.

An explanation of how missing data were handled.

This information can be included in the main text, supplementary information, or relevant data repository. Please note that providing these underlying data is a requirement for publication in this journal, and if these data are not provided your manuscript might be rejected.

***Response:*** Thank you for reviewing our work and providing valuable feedback. We fully acknowledge the importance of including a list of excluded studies to enhance transparency and reproducibility. Excel files containing all relevant data, such as the names of data extractors, extraction dates, eligibility criteria, and the necessary information for replication, are provided in the Supporting Information files. Furthermore, a table summarizing the risk of bias and quality assessments for each study or outcome is included (Table S2). The manuscript also contains a detailed explanation of the methods used to address missing data.

6.The manuscript should be completed by an indept analysis when possible of the frequency of the SNPs in different populations where these data have not been evaluated
***Response:*** Thank you for your valuable feedback on our study. We noted the observed discrepancies between the SNP frequencies in our study and those reported in the HapMap and 1000 Genomes Project databases (Supplemental Table 4). We believe these discrepancies may stem from several factors: First, HapMap primarily reflects population frequencies under broad ethnic classifications, whereas our current study focuses on specific case-control cohorts. Genetic drift, founder effects, and subpopulation differences within ethnic groups may contribute to variations in allele frequencies. Second, the higher T allele frequency observed in our case group supports the role of rs2476601 as a susceptibility locus for type 1 diabetes (T1D), while the slight enrichment in the control group may suggest the presence of undiagnosed autoimmune conditions. Additionally, differences in sample size, genotyping methodologies, and environmental selection pressures could further explain the observed frequency variations. We agree with your suggestion that future studies involving larger and more diverse populations are needed to validate our findings and to further elucidate the genetic basis of T1D susceptibility. We have incorporated these explanations into the Discussion section and addressed additional limitations to enhance the clarity and context of our findings. We sincerely appreciate your constructive comments and thank you again for your insights.

7.The manuscript requires revision by a native english speaker. I would revise the sentence in the abstract 'the findings regarding the relationship between SNPs and T1D susceptibility have been inconsistent'. Studies indeed were 'not conclusive in different populations' while in some such as the Italian or caucasian populations the frequency has been extimated and found significant in diabetics at least for the C1858T SNP.

***Response:*** The manuscript has been thoroughly edited by a native English speaker. The abstract sentence now reads: "Studies investigating the relationship between PTPN22 rs2476601 and T1D risk have consistently demonstrated an association in certain populations, whereas research on rs1310182 has yielded conflicting and less conclusive results. "

Additional grammatical improvements have been made throughout the manuscript, and track changes have been used to mark edits for clarity.

8.The Manuscript sounds interesting and cover all aspects and I would suggest a minor revision to improve the discussion section more with slightly grammarly and English improvment while the data is correctly addressed and conclsuion you can add 2 3 lines more.

***Response:*** We appreciate the positive feedback. The Discussion section has been revised for clarity and grammatical accuracy. We have also expanded the Conclusion section to further emphasize the significance of our findings. Specifically, we added 2–3 lines to highlight the potential implications of our study for understanding genetic contributions to T1D and the importance of population-specific research.

9.PLOS authors have the option to publish the peer review history of their article . If published, this will include your full peer review and any attached files.

***Response:*** We understand this option and will consider publishing the peer review history after the final acceptance of the manuscript.

10.While revising your submission, please upload your figure files to the Preflight Analysis and Conversion Engine (PACE) digital diagnostic tool, <https://pacev2.apexcovantage.com/>. PACE helps ensure that figures meet PLOS requirements. To use PACE, you must first register as a user. Registration is free. Then, login and navigate to the UPLOAD tab, where you will find detailed instructions on how to use the tool. If you encounter any issues or have any questions when using PACE, please email PLOS at [figures@plos.org](mailto:figures@plos.org). Please note that Supporting Information files do not need this step.

***Response:*** We thank you for suggesting that we use the PACE tool to ensure that our data meets the technical requirements of PLOS ONE. Following the instructions provided, we have performed the necessary evaluation and conversion of our graphical files to meet the journal's specifications.

Thank you for your detailed feedback and guidance. We hope that the revised manuscript meets the journal’s requirements. Please do not hesitate to contact us if further modifications are required.

Kind regards,

Fei-fei Wu
