# Supplementary material for: The Association between PTPN22 SNPs and susceptibility to type 1 diabetes: An updated meta-analysis
Source: PLoS One. 2025 Apr 16;20(4):e0321624. doi: 10.1371/journal.pone.0321624 (PMC12002458; doi:10.1371/journal.pone.0321624)
Supplement: Attachment — Submitted filename: Response_to_Editor_auresp_2.docx [file pone.0321624.s011.docx]

Dear PLOS ONE Staff,

On behalf of all co-authors, I sincerely appreciate your constructive feedback and the opportunity to revise our manuscript titled "The Association between PTPN22 SNPs and Susceptibility to Type 1 Diabetes: An updated meta-analysis" (manuscript number: PONE-D-24-50318). We have carefully addressed each point raised in your review and detailed our responses below. Revised sections are marked in the manuscript, and supplementary files have been updated accordingly.

1.Tables 1 and 2 - Zak et al. 2023 examined patients from Armenia, not from America.

***Response:*** We thank the editor for bringing this error to our attention. The geographic origin of the study population in Tables 1 and 2 has been corrected accordingly. Zak et al. (2023) is now accurately described as examining patients from Armenia.

2.Fig 2 - state clearly the reasons for exlusion of studies explicitly mentioned in the figure.

***Response:*** We appreciate the suggestion to improve clarity. In the manuscript, we have clearly outlined the specific reasons for excluding certain studies. These reasons include studies with quality scores ≤12 and studies related to HWD. Additionally, to enhance transparency, we have provided a detailed explanation of the exclusion criteria in the Quality Assessment section of the manuscript

3.Fig. 1 - typo "titlis".

***Response:*** We thank the editor for pointing out this typographical error. The word "titlis" in Figure 1 has been corrected to "titles." Additionally, we have thoroughly reviewed the manuscript and figures to identify and correct any other typographical errors, ensuring the accuracy of the content.

4.The whole study needs to better reflect the geographic gradient of the study polymorphisms within the Caucasian populations. There are enormous differences in the prevalence of the study rs in general and T1DM Caucasian populations both in the N-S and E-W directions throughout Europe. It is described the best for rs2476601. These differences are enormous and prevent generalization of conclusions for the whole Caucasian population.

***Response:*** We thank the editors for their valuable comments regarding the need to consider geographic differences in the prevalence of the studied polymorphisms within Caucasian populations. In response, we have significantly revised the manuscript and Table 3 to address this concern. Specifically:

We excluded mixed-race populations and performed subgroup analyses based on geographic regions (Eastern, Western, Northern, Southern, and Central Europe). Our forest plots and heterogeneity analyses revealed notable regional variations in effect sizes. Heterogeneity was highest in Southern Europe (I² = 63.2%, p = 0.028) and moderate in Western Europe (I² = 59.3%, p = 0.086). In contrast, Eastern, Northern, and Central Europe exhibited minimal heterogeneity (I² = 0.0–10.5%, p > 0.3). The overall analysis demonstrated a significant correlation (z = 8.60, p < 0.001), consistent with findings from most regions. However, the results for Southern Europe were not statistically significant (z = 1.23, p = 0.217), potentially reflecting differences in population structure or allele frequencies.

These findings underscore the importance of accounting for geographic differences when interpreting genetic associations in Caucasian populations and further support the robustness of our conclusions for most regions. We have further discussed these differences and their implications in the revised discussion section of the manuscript.

5.I cannot open the supplementary files. Please double-check their integrity and reupload them.

***Response:*** We apologize for the inconvenience caused by the issues with the supplementary files. The supplementary information file has been re-uploaded with the following adjustments: it has been converted to the Excel 97-2003 (.xls) format for broader compatibility, and its integrity has been verified using LibreOffice 25.2.1 and WPS Office. If the issue persists, we would be happy to provide the files in alternative formats (e.g., PDF or CSV).

Thank you for your detailed feedback and guidance. We hope that the revised manuscript meets the journal’s requirements. Please do not hesitate to contact us if further modifications are required. We look forward to your response.

Kind regards,

Fei-fei Wu
